# The substrate degradome of meprin metalloproteases reveals an unexpected proteolytic link between meprin β and ADAM10

**DOI:** 10.1007/s00018-012-1106-2

**Published:** 2012-09-01

**Authors:** Tamara Jefferson, Ulrich auf dem Keller, Caroline Bellac, Verena V. Metz, Claudia Broder, Jana Hedrich, Anke Ohler, Wladislaw Maier, Viktor Magdolen, Erwin Sterchi, Judith S. Bond, Arumugam Jayakumar, Heiko Traupe, Athena Chalaris, Stefan Rose-John, Claus U. Pietrzik, Rolf Postina, Christopher M. Overall, Christoph Becker-Pauly

**Affiliations:** 1 Institute of Biochemistry, Christian-Albrechts-University, 24118 Kiel, Germany; 2 Institute of Molecular Health Sciences, Swiss Federal Institute of Technology Zurich, ETH Hoenggerberg, HPM D24, Zurich, Switzerland; 3 Departments of Oral Biological and Medical Sciences and Biochemistry and Molecular Biology, Centre for Blood Research, University of British Columbia, Vancouver, BC Canada; 4 Institute of Pharmacy and Biochemistry, Johannes Gutenberg-University, Mainz, Germany; 5 Institute of Physiology and Pathophysiology, University Medical Center, Johannes Gutenberg-University, Mainz, Germany; 6 Cell and Matrix Biology, Johannes Gutenberg University, Mainz, Germany; 7 Institute of Pathobiochemistry, University Medical Center, Johannes Gutenberg-University, Mainz, Germany; 8 Clinical Research Unit, Department of Obstetrics and Gynecology, Technical University of Munich, Munich, Germany; 9 Institute of Biochemistry and Molecular Medicine, University of Bern, Bern, Switzerland; 10 Department of Biochemistry and Molecular Biology, The Pennsylvania State University College of Medicine, Hershey, PA USA; 11 Department of Experimental Therapeutics, M.D. Anderson Cancer Center, The University of Texas, Houston, TX USA; 12 Department of Dermatology, University Hospital Münster, Münster, Germany; 13 Unit for Degradomics of the Protease Web, Christian-Albrechts-University, Rudolf-Höber-Str. 1, 24118 Kiel, Germany

**Keywords:** Meprin, ADAM10, Metalloproteases, Proteomics, TAILS, Degradome

## Abstract

The in vivo roles of meprin metalloproteases in pathophysiological conditions remain elusive. Substrates define protease roles. Therefore, to identify natural substrates for human meprin α and β we employed TAILS (terminal amine isotopic labeling of substrates), a proteomics approach that enriches for N-terminal peptides of proteins and cleavage fragments. Of the 151 new extracellular substrates we identified, it was notable that ADAM10 (a disintegrin and metalloprotease domain-containing protein 10)—the constitutive α-secretase—is activated by meprin β through cleavage of the propeptide. To validate this cleavage event, we expressed recombinant proADAM10 and after preincubation with meprin β, this resulted in significantly elevated ADAM10 activity. Cellular expression in murine primary fibroblasts confirmed activation. Other novel substrates including extracellular matrix proteins, growth factors and inhibitors were validated by western analyses and enzyme activity assays with Edman sequencing confirming the exact cleavage sites identified by TAILS. Cleavages in vivo were confirmed by comparing wild-type and meprin^−/−^ mice. Our finding of cystatin C, elafin and fetuin-A as substrates and natural inhibitors for meprins reveal new mechanisms in the regulation of protease activity important for understanding pathophysiological processes.

**Electronic supplementary material:**

The online version of this article (doi:10.1007/s00018-012-1106-2) contains supplementary material, which is available to authorized users.

## Introduction

Among proteases, the meprin α and β metalloproteases show a unique structure and specificity. Meprin α is the largest extracellular secreted protease, building chain and ring-like structures up to 6 MDa in size [1, 2]. In comparison, meprin β is a membrane-bound dimer, but can be released from the cell surface by shedding [3]. However, like many proteases, their in vivo roles remain unclear through lack of understanding of their substrate repertoires, also known as the substrate degradome [4]. Meprins have been implicated in connective tissue formation, angiogenesis and immunology, but like many proteases, they are also potentially important in pathologies. Thus, meprins are associated with inflammatory bowel disease, kidney nephritis, fibrosis and cancer [5–7], but their substrates and roles in these diseases remain unclear.

To date meprin metalloproteases have been shown to hydrolyze a number of different proteins in vitro [8], but only a few in vivo substrates have been identified, such as interleukin-1β [9], interleukin-18 (IL-18) [10], transforming growth factor α (TGF-α) [11] and vascular endothelial growth factor A (VEGF-A) [12]. Recently, high-throughput approaches involving protease proteomics (also known as degradomics) have been applied to meprins. Proteomic identification of cleavage site specificity [13] shows that meprins preferentially cleave N-terminal to the acidic amino acids glutamate and aspartate [14]. Indeed, this is concordant with most of the few known substrates. For example, procollagen III is processed by meprin α and β at an aspartate in P1′, releasing the mature triple helical collagen, which then further assembles to form collagen fibrils [15]. Meprin β specifically cleaves in the N-terminal region of the amyloid precursor protein (APP), again at negatively charged amino acid residues [16], releasing two peptides that have been identified in the brain cerebrospinal fluid of patients with Alzheimer’s disease [17]. While this implicates meprin β in neurological pathophysiology, the lack of known substrates reveals yawning gaps in mechanistic information on meprin function in vivo.

Proteases rarely act alone. Instead, many form activation networks or cascades [18–20]. Indeed, it has been suggested that proteases form a deeply interconnected protease web embedded in every tissue proteome [21–24]. In this network, inhibitors are important control points in proteolytic signaling, such as the tissue inhibitors of matrix metalloproteases (TIMPs) 1–4 for MMPs and TIMP3 for ADAMs (a disintegrin and metalloprotease domain-containing protein), yet no specific natural inhibitors have been identified for the meprins. In another prominent example, the serine protease inhibitor lymphoepithelial Kazal-type-related inhibitor (LEKTI) has a crucial impact on the homeostasis of epidermal skin as shown from mutations in the cognate *SPINK5* gene. Such mutations result in nonfunctional LEKTI incapable of blocking kallikreins (KLK) and KLK-related peptidases, so leading to severe defects in skin desquamation in Netherton syndrome mediated by hyperactivity of KLK-related peptidases [25]. In the protease web, protease inhibitors can be inactivated by proteolytic activity from other classes of proteases. For example, the cysteine protease inhibitor cystatin C is cleaved and inactivated by MMPs leading to increased cathepsin L activity [26].

Another family of metalloproteases, the ADAMs, are cell surface proteases often involved in protein shedding from the plasma membrane [20, 27]. ADAM10 is important for the development of blood vessels and the central nervous system, as well as in pathological conditions such as inflammation and cancer [28, 29]. Recently, it was shown that ADAM10 is the major sheddase of notch receptors, involved in the release of the extracellular domain and so mediating skin development [30]. As the constitutive α-secretase of amyloid protein [31, 32], ADAM10 is presumed to prevent the formation of aggregates of neurodegenerative amyloid β peptides derived from the amyloid precursor molecule by cleavage by β- and γ-secretases [33].

Although many studies have demonstrated physiologically relevant regulation of ADAM10 activity by TIMPs [34], little is known about the initial activation of ADAM10 [32]. While the proprotein convertase furin cleaves the propeptide proADAM10 at the maturation site RKKR in the secretory pathway, the globular propeptide remains noncovalently bound to the active site thereby still inhibiting the protease [35]. Thus, how ADAM10 gains catalytic competence in vivo by complete removal of the propeptide is unknown, but this is an important question in the understanding of Alzheimer’s disease where the α-secretase activity is outweighed by pathological cleavage by β- and γ-secretases [18, 32].

In the present work, terminal amine isotopic labeling of substrates (TAILS) [36, 37] was used to identify the cleavage sites of native protein substrates of meprins α and β by N-terminal peptide enrichment and proteomic analyses. We identified physiologically relevant meprin substrates in the cellular context, which is important for identifying physiologically relevant targets [21, 24, 38, 39]. Of the 151 substrates identified with high confidence, one of the most interesting was cleavage in the propeptide of ADAM10 by meprin β, potentially leading to propeptide destabilization and release from the catalytic domain, so completing activation. We also describe a broad range of protease inhibitors that are cleaved by meprins including LEKTI, implicating meprins in the indirect regulation of KLK activity. Finally, we found several natural inhibitors of meprins that we propose as important for in vivo regulation of these two proteases.

## Materials and methods

### Protein expression and purification of meprin, APP and proADAM10

Human meprin α and β were expressed and purified according to previously published methods [2, 40]. APP695 and APP751 were produced as described in previously [16]. A truncated version of murine proADAM10 was engineered for recombinant expression lacking the ADAM10 signal peptide and regions C-terminal of the protease domain using the following primers:

- Sense: 5′-CATGCCATGGGGAGGTCAGTATGGAAATCCTTTAAATAAATATATTAGACATTATGAAGG-3′
- Antisense: 5′- CCGCTCGAGGATAGGCTGGCCAGATTCAACAAAACAGTTGTTCCTCTTCTTCTCAAGCAC -3′

Constructs were ligated into pFastBac (Gibco) containing the meprin β signal peptide, followed by a 6× His-tag, resulting in the expression of soluble proADAM10. Primers were synthesized by Invitrogen GmbH and sequences of constructs were verified by DNA sequencing (GENterprise GmbH).

Recombinant protein was expressed using the Bac-to-Bac expression system (Gibco) following the manufacturer’s instructions. All media and supplements were obtained from Gibco. Recombinant baculoviruses were amplified in adherently growing *Spodoptera frugiperda* (Sf-9) insect cells at 27 °C in Grace’s insect medium supplemented with 10 % fetal bovine serum, 50 units/ml penicillin and 50 μg/ml streptomycin. Protein was expressed in 500 ml suspension cultures of BTI-TN-5B1-4 (HighFive) insect cells growing in Express Five SFM supplemented with 4 mM glutamine, 50 units/ml penicillin and 50 μg/ml streptomycin in Fernbach flasks using a Multitron orbital shaker (Infors AG). Cells were infected at a density of 2 × 10^6^ cells/ml and protein expression was stopped after 72 h; media were stored at −20 °C until further use. Recombinant APP was further purified from the media by ammonium sulfate precipitation (60 % saturation), stirring overnight at 4 °C, followed by centrifugation at 11,000 *g* for 2 h at 4 °C. Pellets were dissolved in 1/10 volume of 50 mM NaH_2_PO_4_, 300 mM NaCl, pH 8.0, and dialyzed against a solution containing 50 mM NaH_2_PO_4_, 300 mM NaCl and 10 mM imidazole, pH 8.0, and then the protein solution was loaded on a Ni–NTA column. After a washing step using a solution containing 50 mM NaH_2_PO_4_, 300 mM NaCl and 25 mM imidazole, pH 8.0, protein was eluted with the same buffer containing 50 mM imidazole, pH 8.0. ProADAM10 was analyzed by sodium dodecyl sulfate polyacrylamide gel electrophoresis (SDS-PAGE), western blot (monoclonal Penta His 1:1000; Qiagen), and MALDI-TOF at the Institut Fédératif de Recherche (IFR) 128 (Lyon, France).

### Cell culture

HaCaT, HEK293, Caco2 and U373 cells were grown each in Dulbecco′s modified Eagle’s medium (DMEM) GlutaMAX (Invitrogen) supplemented with 5 % calf serum until they reached approximately 60–80 % confluence. Caco2 and U373 cells were transiently transfected with full-length meprin β-pIRES2-EGFP cDNAs with the Nanofectin transfection reagent (PAA Laboratories GmbH) according to the manufacturer′s instructions. Transfections were carried out for 24 h, after which all cell cultures were washed with serum-free and phenol-free DMEM (Invitrogen). Nontransfected cells were treated with 5 nM recombinant meprin α or 5 nM recombinant meprin β. Cells were incubated for 24–72 h, depending on viability, in serum-free and phenol-free DMEM either as transfectants, with recombinant enzyme or not treated. Samples of transfected cell medium and lysate were used for detection of meprin β by using a polyclonal anti-meprin β antibody [2]. Signal detection in all western blotting experiments was carried out by enhanced chemiluminescence (ECL). For expression of proADAM10, HEK293 monolayers (70–80 % confluence) were transiently transfected with full-length proADAM10-pc DNA or pc DNA as mock controls as described above. Culture supernatant was collected 24 h after transfection.

### Collection of cell media

After removal of secretome for TAILS analysis, the following protease inhibitors were added: 1 μM E-64 (l-trans-epoxysuccinyl-leucylamido-(4-guanidino)butane), 1 mM ethylenediaminetetraacetic acid (EDTA) and 0.5 mM phenylmethanesulfonylfluoride (PMSF). To remove cells and cell debris, the medium was centrifuged for 5 min at 500 *g* and the supernatant for 30 min at 8,000 *g*. Clarified secretome was 10× concentrated using 3 K centrifugal filter units (Millipore), simultaneously exchanging the buffer with 10 mM HEPES, pH 7.5. The Bradford assay was used to determine protein concentrations.

### TAILS

Cells were grown in DMEM, 5 % calf serum to 70 % confluence, washed extensively to remove serum proteins, and grown overnight in serum-free medium. Cells were washed again, incubated in phenol red-free, serum-free medium and incubated with recombinant human meprin α or β. Conditioned medium proteins were harvested at 48 h when the cells were between 80 and 90 % confluence. Protease inhibitors (1 mM EDTA, 1 mM PMSF) were immediately added and the medium clarified by centrifugation (5 min, 500 *g*), filtration (0.22 μm) and additional centrifugation (30 min, 8,000 *g*). The proteins were 100× concentrated by ultrafiltration using Amicon Ultra-15 centrifugal filter units (3 kDa cut-off; Millipore). The sample buffer was changed to 50 mM HEPES, 150 mM NaCl and 10 mM CaCl_2_ by five cycles of dilution and concentration in the same concentrating device. Protein concentrations were determined using the bicinchoninic acid (BCA) assay (Pierce) and the Bradford assay (BioRad). The TAILS procedure including isotopic labeling, tryptic digestion, amine-terminal blocked peptide enrichment, liquid chromatography-MS/MS, data analysis and peptide abundance ratio was performed as previously described [36]. MS2 spectra were searched against the human International Protein Index protein database (v.3.42; 72,346 protein entries) using Mascot version 2.2 (Matrix Science) or X! Tandem (2007.07.01 release). The following parameters were applied: semi-ArgC cleavage specificity with up to two missed cleavages, cysteine carbamidomethylation and peptide lysine iTRAQ as fixed modifications, and N-terminal iTRAQ, N-terminal acetylation and methionine oxidation as variable modifications. Tolerance for precursor and fragment ions was set at 0.4 Da, and the ESI-QUAD-TOF scoring scheme was used. Secondary validation was performed using the Trans Proteomic Pipeline (TPP v. 4.2, rev. 0, build 200811181145) [41, 42] using the PeptideProphet [43] and iProphet [44] algorithms for peptide/protein assignment, and iTRAQ reporter ion intensities were quantified using Libra. Peptides were filtered by iProphet scoring, and only assignments with a probability ≥0.95 were included in further analysis. Multiple spectra were merged for individual peptides, and reporter ion intensity ratios were calculated by intensity-dependent weighted averaging using statistical models described previously [36, 45]. Accordingly, peptides with a protease/control iTRAQ ratio of ≥10 were considered as only present in the protease-treated sample and thus derived from the activity of the test protease. Peptides with a ratio of less than ten require biochemical validation to confirm that they represent cleavage sites in native protein substrates.

### Primary murine fibroblast isolation

Tissue biopsies from the ears of 6-week-old meprin β^−/−^ and wild-type mice were excised, cut into small pieces using a sterile scalpel, and washed in 70 % ethanol for 2 min. After incubation in 0.05 % trypsin for 48 h at 4 °C, pieces were transferred to 25-cm^2^ cell culture flasks coated with fetal calf serum coated, and after 48 h of incubation cultured in DMEM supplemented with 10 % (vol/vol) fetal calf serum and 50 μg/ml gentamicin. Ear pieces were incubated every 48 h with 0.05 % trypsin until the first cells adhered. The medium was changed after 48 h and the cells cultured until confluence.

### Generation of meprin β-expressing cells

HEK293 cells were transfected with plasmid pIRES2-EGFP-meprin-β-HA using lipofectamine 2000 (Invitrogen) and selected via G418 to generate stable cell lines. Selected cells stably expressed C-terminal HA-tagged human meprin β and EGFP.

### Meprin β cleavage assays and inhibitor treatments

Cells were seeded into six-well plates coated with poly-l-lysine. For stimulation, mouse embryonic fibroblasts (MEF) and HEK cells were grown to 80–90 % confluence. Cells were washed twice with serum-free DMEM and then secretion medium (serum-free DMEM supplemented with 2 mM glutamine) was added. For induction of shedding, either phorbol 12-myristate 13-acetate (PMA, 1 μM) or A23187 (1 μM) was added for 3 h. MEF were then analyzed by flow cytometry. Experiments in the presence of metalloprotease inhibitors (GM6001, 25 μM; GI254023X, 25 μM or 100 nM) were performed by preincubating HEK cells with inhibitor in secretion medium for 1 h at 37 °C and then PMA or A23187 was added for 3 h. In control experiments, the solvent DMSO was present in all incubation steps. After the appropriate incubation time, cell culture supernatants were collected, centrifuged for 10 min at 660 *g* and proteins precipitated with 10 % trichloroacetic acid at 4 °C. Proteins were analyzed by western blotting. For comparative and quantitative analysis, solvent-treated cells were used as controls with the effects observed set to 100 %.

### SDS-PAGE and western blot analysis

SDS-PAGE was performed according to standard procedures in 10, 12 or 16 polyacrylamide gels. Coomassie brilliant blue was used for background-free gel staining [46]. For immunoblot analysis proteins were subjected to electrophoresis under reducing conditions and then transferred onto a PVDF membrane (Immobilon P; Millipore) by western blotting. For detection with polyclonal antibodies, the membrane was saturated with 5 % dried milk in Tris-buffered saline for 1 h, incubated with the first antibody for 1 h and subsequently with horseradish peroxidase-conjugated antirabbit IgG (1:10 000) for 1 h at room temperature. Proteins were detected using Rotilumin (Roth) following the manufacturer’s instructions using X-ray film (Hyperfilm ECL; Amersham Pharmacia Biotech). For detection with monoclonal antibodies (1:1,000), the membrane was blocked with 3 % bovine serum albumin and incubated with the appropriate antibody. The secondary antibody (antimouse, coupled with horseradish peroxidase, 1:10,000) was added for 1 h, and ECL (Millipore) was used for subsequent detection. For detection of total murine ADAM10 protein in fibroblasts, a polyclonal ADAM10 antibody directed to the C-terminus was applied [47].

### Processing of recombinant proteins by meprin α and β

The substrates were incubated with 50–100 nM recombinant meprin α or β at 37 °C in 50 mM HEPES, pH 7.5. ProMMP1, LEKTI and KLK7 were expressed and purified as described previously [48–50]. Other proteins were purchased as follows: IGFBP-3 (Immunotools), VEGF-A_165_ (Cell Signaling), syndecan-4 (R&D Systems), cystatin C (BioVendor), elafin (ENZO Life Sciences), fetuin-A (Sigma), secretory leukocyte protease inhibitor (SLPI; R&D Systems), stratifin (Sigma) and desmoglein-1 (R&D Systems). Assay reaction products were subjected to SDS-PAGE and either stained with coomassie blue or analyzed by western blotting. The following antibodies were used: anti-FGF-19 (Bio Vision; 1:1,000, polyclonal), anti-IGFBP-3 (Immunotools; 1:1,000, polyclonal), anti-VEGF-A (Santa Cruz Biotechnology; 1:200, monoclonal), anti-N-APP (Thermo Scientific; 1:200, polyclonal), anti-His-tag (Qiagen; 1:1,000, monoclonal), anti-LEKTI (H-300; Santa Cruz Biotech; 1:200, polyclonal), anti-MMP1 (Abcam; 1:1,000, polyclonal), anti-stratifin (Firma; 1:1,000, monoclonal), and anti-ADAM10 (Abcam; 1:1,000, polyclonal).

### Identification of cleavage sites

For N-terminal sequencing, proteins were separated by SDS-PAGE, blotted onto PVDF membranes for Edman degradation, stained with coomassie brilliant blue, and then analyzed at the Protein Micro-sequencing Centre of the Institut Fédératif de Recherche (IFR) 128 (Lyon, France).

### Inhibition studies

Inhibition of meprin by elafin was compared with activity after incubation with fetuin-A and cystatin C used as positive controls [51]. The inhibition assay of meprin α and β was performed using 5 × 10^−5^ M elafin. The inhibitory potential of SLPI (1 μM) and LEKTI (100 nM) after meprin cleavage (100 nM and 10 nM, respectively) was demonstrated by the analysis of (chymo)tryptic substrates. Following incubation of inhibitors with meprin α or β for 30 min at 37 °C, meprin activity was blocked by the addition of 200 nM of the specific inhibitor actinonin for 15 min at 37 °C. The inhibitory capacity of SLPI against KLK7 was determined using the quenched fluorogenic substrate Suc-LLVY-AMC (Bachem) at a final concentration of 100 μM. KLK2 activity was detected using Z-Pyr-G-R-AMC (Peptanova) at a final concentration of 100 μM. The enzyme activity was measured with a Synergy™ HT reader (BioTek). The proteolytic activity was determined in relation to the emission at 405 nm with excitation at 320 nm. The activity was determined from the slope of the initial linear range of the curve.

### Activity assays using fluorogenic peptides to validate catalytic properties of proADAM10 and meprin β

To test the enzymatic efficiency of proADAM10 and ADAM10 activated by meprin β and meprin β alone, quenched fluorogenic peptide substrates were used: Mca-KPLGLA2pr(Dnp)AR-NH_2_ for proADAM10 (Peptanova) and Mca-YVADAPK(Dnp)-NH_2_ for meprin β (Peptide Institute Inc.) which was used at a final concentration of 10 μM. The enzyme activity was measured with a Varioskan Flash fluorescence spectrometer (Thermo Scientific). Data were analyzed using SkanIt Software 2.4 for Varioskan Flash. Enzymes were buffered in 50 mM HEPES, pH 7.5, and proADAM10 was used at a final concentration of 10 μM and meprin β at 15 nM. The ADAM10 inhibitors GI254023X and GM6001 were added each at a final concentration of 10 μM and preincubated for 20 min at room temperature. ADAM activity in cell culture supernatants from murine fibroblasts was measured at 37 °C, and the fluorescence was detected every 12 s for 120–240 min. Proteolytic activity was determined in relation to the emission at 405 nm with excitation at 320 nm. The activity was determined from the slope of the initial linear range of the curve.

### Quantitative real-time PCR

Total RNA was isolated from primary fibroblasts of wild-type and meprin β knockout mice (*n* = 3) according to the instructions with the GeneJET RNA purification kit (Fermentas, Thermo Scientific) and transcribed into complementary DNA using RevertAid™ transcriptase (200 U/ml), 10 mM nonspecific oligo d(T) primers, and 200 mM dNTPs (Fermentas, Thermo Scientific). The cDNA obtained was subjected to quantitative real-time PCR using a LightCycler480^®^ real-time PCR system (Roche Applied Science). Amplification reaction consisted of a hold of 10 min at 95 °C and 45 cycles (10 s/95 °C, 30 s/60 °C). For assay design the Universal ProbeLybrary system (http://qpcr.probefinder.com/roche3.html) was used to amplify intron spanning regions for the gene of interest. Relative amounts of target gene mRNA were normalized to the housekeeping gene GAPDH. The following primers/probes were used:

- ADAM10 sense: 5′-gggaagaaatgcaagctgaa-3′
- ADAM10 antisense: 5′-ctgtacagcagggtccttgac-3′

ΔΔCp values were used to calculate the relative expression for each data point.

### Flow cytometry

Tumor necrosis factor-α converting enzyme (TACE) knockout MEF cells (5 × 10^5^), untreated or stimulated with PMA or A2318, or DMSO as control, were detached from the cell culture plate with Accutase (PAA Laboratories GmbH) and washed twice with FACS buffer (1 % BSA in PBS). Following centrifugation at 1,000 *g* for 5 min at 4 °C, cells were blocked with 10 % FCS in PBS for 15 min. After pelleting, cells were incubated with a polyclonal meprin β antibody (1:1,000) in FACS buffer for 60 min on ice. Cells were then washed once in FACS buffer and incubated with Alexa Fluor 647-conjugated goat antirabbit antibody for 30 min on ice. After a final wash, cells were resuspended in FACS buffer and analyzed by flow cytometry (FACS Canto; Becton–Dickinson, Heidelberg, Germany). All assays were carried out in triplicate. Data were analyzed using FCS Express v. 3 (De Novo Software, Los Angeles, CA).

### Animal and tissue preparation

Muscle, kidney, intestine and hippocampus from wild-type and meprin knockout animals were isolated as previously described in [16].

### Supplementary data

Supplementary data are available online from Cellular and Molecular Life Sciences journal at www.springer.com.

## Results

### Proteomics analysis of proteins cleaved by meprin α and β in the cellular context

**Fig. 1:**
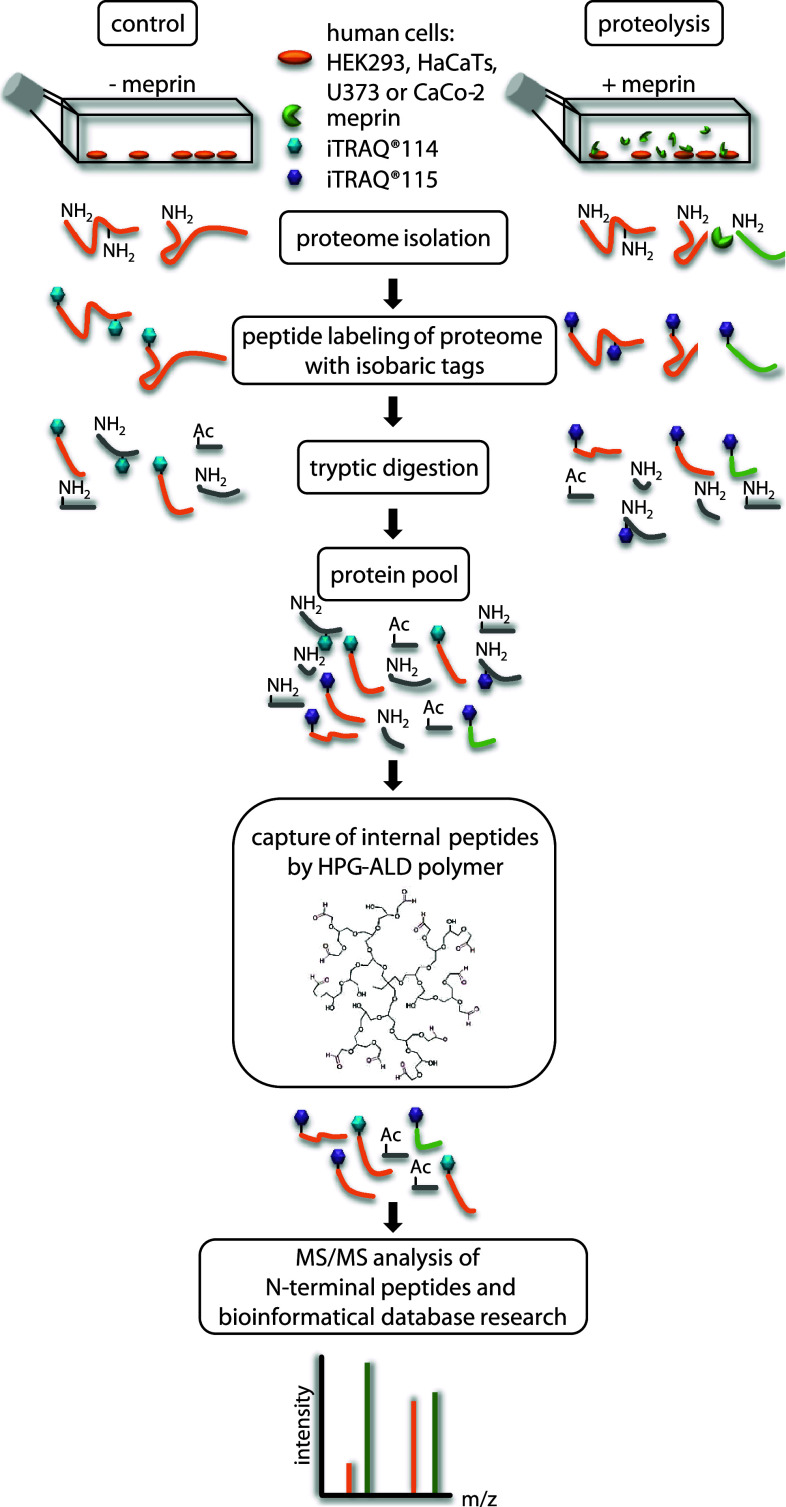
TAILS workflow for proteomic meprin α and β substrate screening. The serum-free proteomes of cells of the human cell lines HEK293 (human embryonic kidney), HaCaT (human adult low-calcium high-temperature), U373 (glioblastoma) and Caco2 (colon cancer) treated with recombinant soluble meprin α or β or transiently transfected with full-length meprin β were collected and compared to those of control cells. N-termini of the whole isolated proteins were labeled with iTRAQ reagents. Following tryptic digestion internal peptides were blocked and removed by polyaldehyde dendritic polymer and N-termini (natural or iTRAQ-labeled) were enriched for liquid chromatography on a HPLC system. Labeled peptides were analyzed by MS/MS with *m*/*z* peaks of 114 and 115. Cleavage sites were often validated by Edman sequencing. iTRAQ quantification allowed up to eight samples to be analyzed simultaneously using eight isobaric tags. *HPG* hyperbranched polyglycerols, *ALD* HPG aldehydes

To identify extracellular substrates of meprin α and β, we analyzed the secretome of HaCaT, HEK293, Caco2 and U373 cells incubated with recombinant meprin α and β. We also analyzed secretomes of HaCaT, Caco2 and U373 cells that had been transfected to express full-length membrane-bound meprin β. As a control, HEK293 cells were treated twice with recombinant meprin β to validate reproducibility (Fig. 1, Online resource 1). By MS/MS (tandem mass spectrometry) using an ion cut-off ratio of 3.0, we identified with high confidence 151 new extracellular N-terminal peptides, of which 117 had a ratio of ≥10.0, as extracellular substrates of meprin α and β. The most promising candidates were classified into six categories.

### Other proteases

**Fig. 2:**
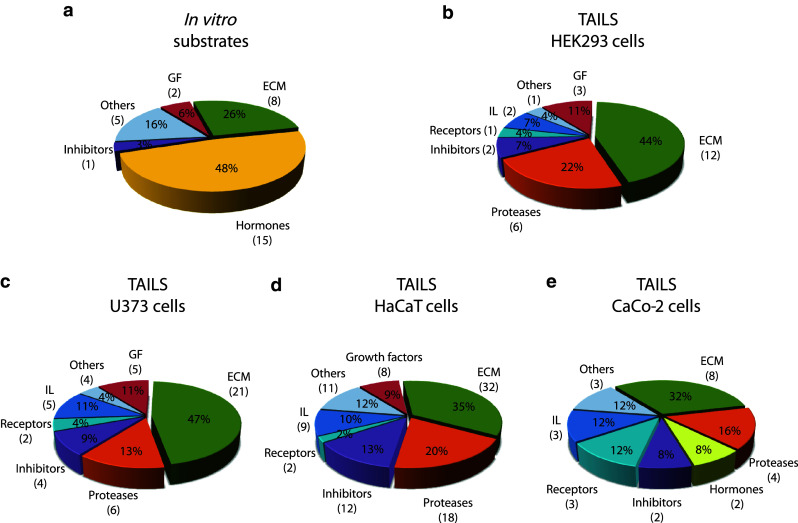
Classification of meprin substrates identified by TAILS. To date, the majority of known substrates for meprins is from analysis of isolated proteins in vitro (**a**). By proteomic screening of cells of the human cell lines HEK293 (**b**), U373 (**c**), HaCaT (**d**) and Caco2 (**e**) novel substrates were identified including inhibitors, receptors, proteases and other proteins with uncharacterized functions. Categories were determined based on UniProt database entries. Numbers of identified substrates are given in parentheses. *IL* immunological, *GF* growth factor, *ECM* extracellular matrix

**Fig. 3:**
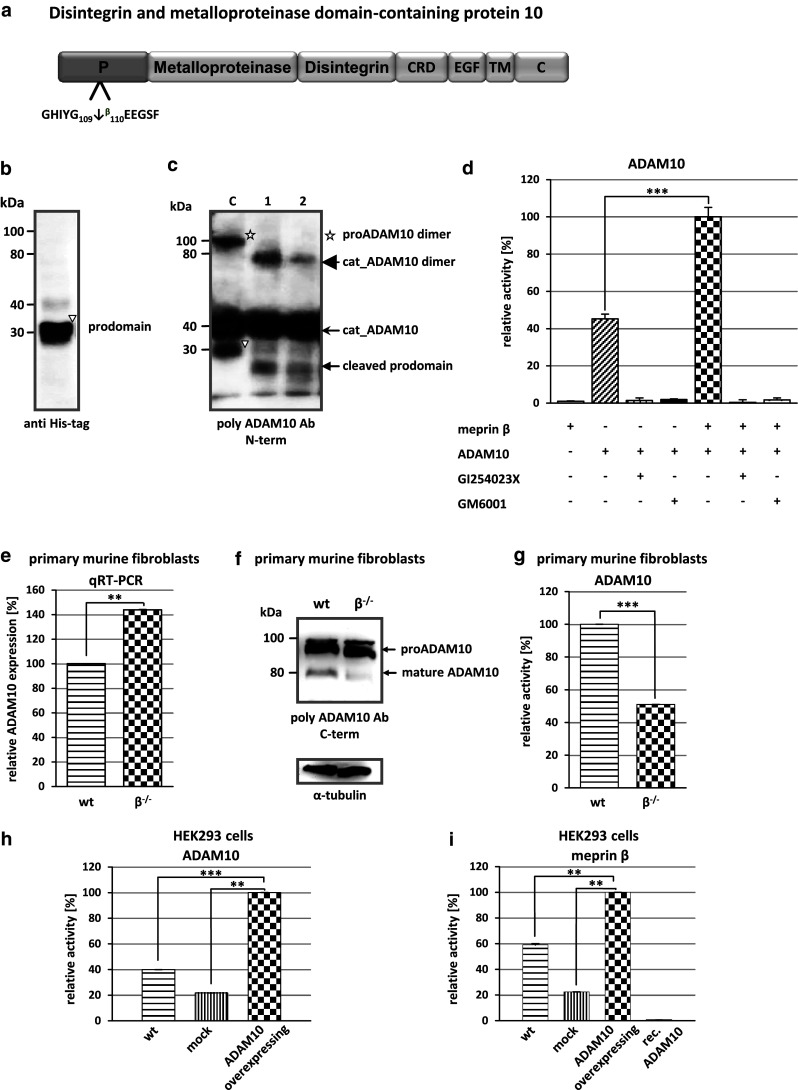
Activation of proADAM10 by meprin β. **a** Schematic structure of ADAM10 indicating the cleavage site identified by TAILS for meprin β. Cleavage occurs within the propeptide of ADAM10 between Gly109 and Glu110. *CRD* cysteine-rich domain, *EGF* epidermal growth factor-like domain, *TM* transmembrane domain, *C* cytoplasmic tail. **b** Recombinant proADAM10 was expressed in insect cells and purification was facilitated using an N-terminal His-tag. Purified proADAM10 was subjected to SDS-PAGE and analyzed by western blotting with an anti-His-tag antibody, detecting the prodomain of ADAM10 (*triangle*). **c** Recombinant proADAM10 was proteolytically processed by meprin β at 37 °C for 1 and 5 min (*asterisk* proADAM10 dimer). After incubation with meprin the propeptides are cleaved (*cat_ADAM10*; *arrowhead*, *triangle* prodomain). **d** The relative activity of ADAM10 was monitored using the FRET peptide Mca-KPLGLA2pr(Dnp)-AR-NH_2_. The ADAM10 concentration was 10 μM and 15 nM for meprin β, inhibitors GI254023X and GM6001 were used at a final concentration of 1 μM. **e**, **f** Quantitative real-time PCR (qRT-PCR) and western blotting were used to detect ADAM10 expression in wild-type (*wt*) and meprin β knockout (*β* ^*−/−*^) fibroblasts. **g** Primary murine fibroblasts of meprin β^−/−^ mice show a reduced ADAM10 activity compared to their wild-type counterparts measured using the ADAM10 fluorogenic substrate (*wt* wild-type). **h** HEK293 cells were transiently transfected with full-length ADAM10 cDNA and activity was determined by the ADAM10 FRET substrate. Untransfected wild-type and mock-transfected cells were used as controls. **i** Meprin β activity is increased fivefold in ADAM10-overexpressing HEK293 cells determined by the meprin β-specific FRET peptide Mca-YVADAPK(Dnp)-NH_2_. Untransfected wild-type and mock-transfected cells were used as controls. For quantification all experiments were performed in quadruplicate. Significance was determined by the *t* test (**p < 0.05; ***p < 0.01)

Interestingly, a large number of substrates identified by TAILS were proteases (Fig. 2). ADAM9 and ADAM10, commonly known as α-secretase candidates in nonpathological APP processing [52], were both specifically cleaved by meprin α and β. Special regard was paid to ADAM10 where meprin β-transfection of Caco2 cells (Table 1) led to cleavage between Gly109 and Glu110 within the 194 amino acid propeptide (Fig. 3a). To validate this, recombinant proADAM10, N-terminally tagged with six histidines and lacking the C-terminal region downstream of the protease domain to ensure solubility, was produced by heterologous expression in baculovirus-infected insect cells and subsequently purified by Ni-NTA chromatography (Fig. 3b). Western blot analysis of the purified proADAM10 domain and MALDI-TOF analysis verified the identity of the protein revealing the full-length ADAM10 dimer, the catalytic domain (40 kDa) and the prodomain (30 kDa) (Fig. 3b, c). Recombinant proADAM10 was processed by meprin β and yielded in a decrease in the mass of the 100-kDa proADAM10 dimer and the 30-kDa propeptide resulting in minor molecular weight fragments indicating a meprin β-mediated cleavage event (Fig. 3c). As analyzed using the fluorescence resonance energy transfer (FRET) peptide Mca-KPLGLA2pr(Dnp)AR-NH_2_, we found a significantly increased activity of ADAM10 after meprin β cleavage (Fig. 3d). The activity of proADAM10 and meprin β-activated ADAM10 was in both cases fully inhibited using the hydroxamate inhibitors GI254023X and GM6001 at concentrations of 10 μM specific for ADAM inhibition [53]. In assessing the biological relevance of this cleavage, the activity of ADAM10 was reduced from 100 % to 51 % in primary murine meprin β^−/−^ fibroblasts compared to meprin β-expressing control cells (Fig. 3g). Interestingly, while the expression of ADAM10 measured by quantitative real-time PCR in wild-type cells was significantly lower than in meprin β^−/−^ fibroblasts (Fig. 3e), the total protein level of ADAM10 was not dramatically different, although the mature form of ADAM10 was slightly lower in meprin beta^−/−^ cells (Fig. 3f).

**Table 1:**
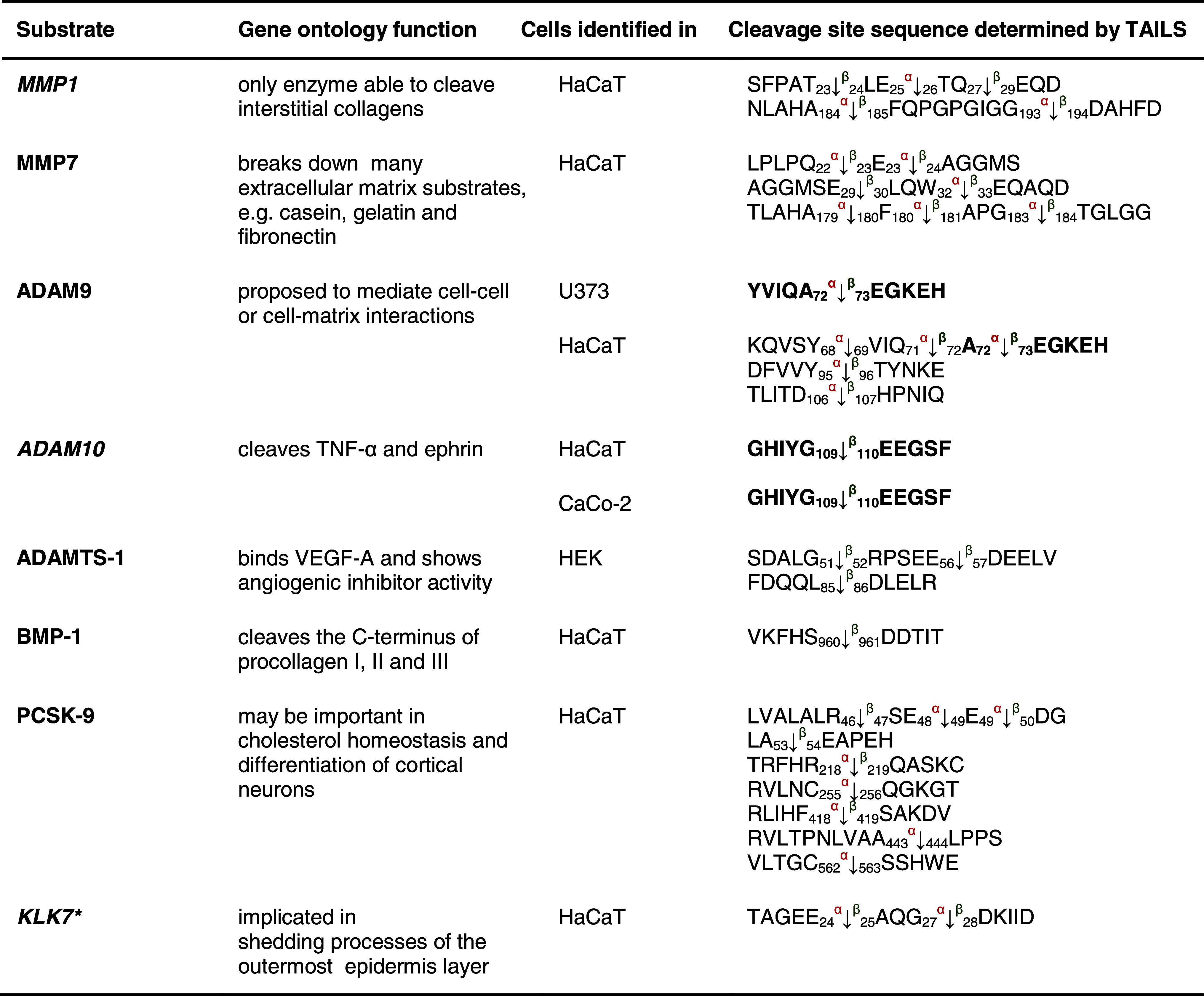
Protease substrates for meprin α and β identified by TAILS

Proteins were analyzed from cell culture media as indicated in the third column after 24 and 48 h, respectively. Caco2 cells were additionally transfected with full-length meprin β cDNA before the conditioned culture medium was collected. Identified substrates presented here are members of the metalloprotease family or belong to the serine proteases. Proteins in *italic* have been further validated in vitro (Fig. 3). Sequences are given in the *one letter code*. *Arrows* indicate the cleavage sites analyzed by MS/MS for meprin α (*α*) and meprin β (*β*). *Asterisks* indicate substrate cleavage sites validated by Edman sequencing.* Numbers* indicate the position of amino acids in full-length protein. Confirmed cleavage sites in more than one secretome are indicated in *bold*.

*MMP* matrix metalloprotease, *ADAM* disintegrin and metalloprotease domain-containing protein 9, *ADAMTS-1* disintegrin and metalloprotease with thrombospondin motifs, *BMP* bone morphogenetic protein, *PCSK* proprotein convertase subtilisin/kexin type 9, *KLK* kallikrein, *TNF* tumor necrosis factor.

**Fig. 4:**
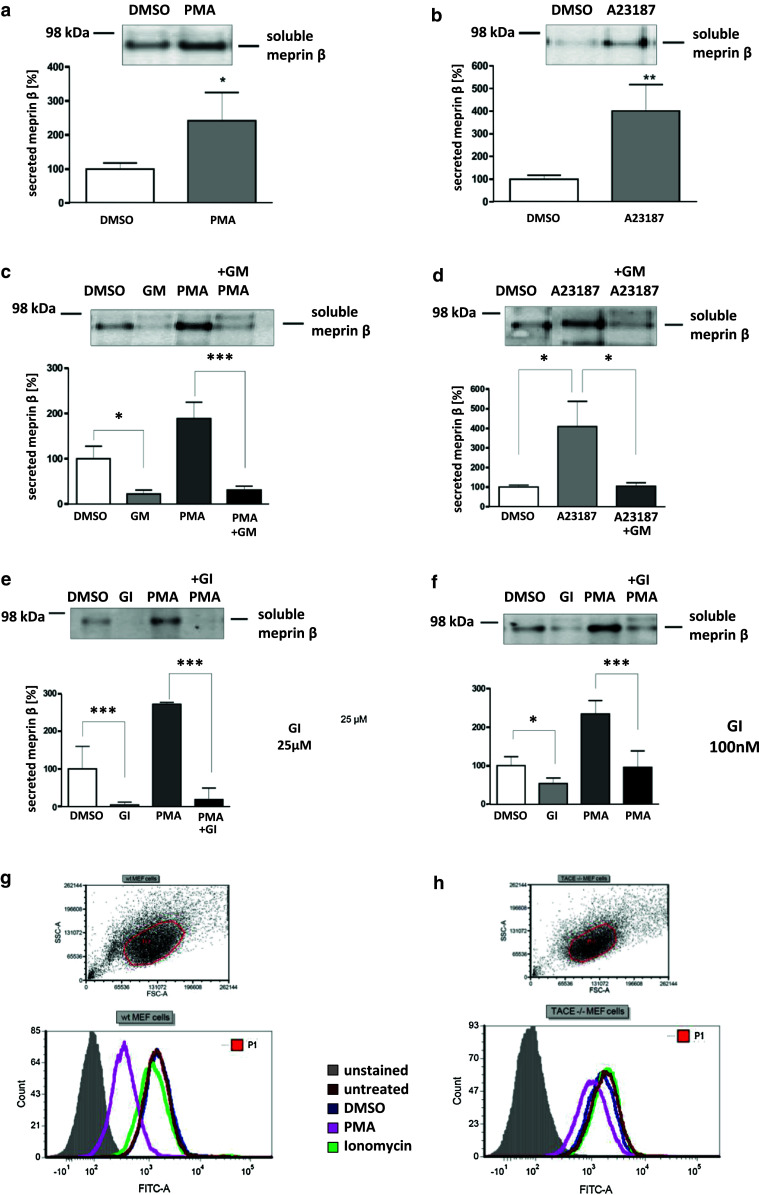
Effects of ADAM10 on ectodomain shedding of meprin β. **a**, **b** Stable meprin β-overexpressing HEK 293 cells were incubated for 3 h with PMA (1 μM, **a**), A23187 (1 μM, **b**) or DMSO as control. The cell culture supernatants were then collected and the proteins precipitated. Soluble meprin β was detected by western blotting using a polyclonal antimeprin β antibody, followed by an antirabbit antibody labeled with horseradish peroxidase and ECL. For quantification, the experiments were performed in quadruplicate. The mean + SD effects are indicated; significance was determined by Student’s unpaired *t* test (*P < 0.05, **P < 0.01). **c**, **d** Meprin β-expressing HEK 293 cells were preincubated for 1 h with GM6001 (25 μM) or DMSO as control, then PMA (1 μM, **c**) or A23187 (1 μM, **d**) was added for 3 h. Soluble meprin β was detected by western blot analyses as described above. For quantification the experiments were done in triplicate. The mean + SD effects are indicated; significance was determined by the one-way ANOVA Bonferroni test (*P < 0.05, ***P < 0.001). **e**, **f** Meprin β-expressing HEK293 cells were preincubated for 1 h with GI254023X (25 μM, **e**. or 100 nM, **f**) or DMSO as control, then PMA (1 μM) was added for 3 h. Soluble meprin β was detected by western blotting. For quantification the experiments were done in triplicate. The mean + SD effects are indicated; significance was determined by the one-way ANOVA Bonferroni test (*P < 0.05, ***P < 0.001). Secretion of meprin β in all tests is indicated relative to the DMSO control. **g**, **h** Membrane-bound meprin β levels were analyzed by flow cytometry in wild-type and TACE (ADAM17) knockout cells incubated with DMSO (1 μM) as control, PMA (1 μM) or ionomycin (A23187, 1 μM). A shift to the left indicates reduced meprin β levels at the cell surface

It has previously been demonstrated that ADAM17 (TACE) is a sheddase for human meprin β [3]. We determine whether ADAM10 is not only activated by meprin β but might also shed meprin β from the cell surface. In HEK293 cells transiently transfected with ADAM10 (Fig. 3h) we demonstrated an increase in meprin β activity in the supernatant of 40 % (Fig. 3i). To confirm that ADAM10 was a constitutive sheddase of membrane-bound meprin β in a cellular system (Fig. 4), we induced endogenous ADAM10 expression by PMA (Fig. 4a) and the calcium ionophore A23187 (Fig. 4b). This resulted in a significant threefold enhancement of soluble meprin β in the supernatant as determined by western blotting using a polyclonal antibody specific for meprin β (Fig. 4a, b). Correspondingly, PMA induction resulted in reduced levels of membrane-bound meprin β expression in cells with or without ADAM17 activity (Fig. 4g, h). Probably due to low expression of endogenous meprin β in these cells, the calcium ionophore ionomycin (A23187) did not significantly induce ectodomain shedding. Meprin β shedding was potently blocked by the inhibitors GI254023X and GM6001 at nanomolar concentrations in cells in both the presence and absence of PMA and A23187 (Fig. 4c–f). Expression of membrane-bound meprin β was not affected by PMA or A23187 stimulation as detected in the cell lysates. Thus, meprin β finalizes the activation of ADAM10 by a destabilizing cleavage in the propeptide of ADAM10 that releases the furin-cleaved propeptide from the catalytic domain. In turn, activated ADAM10 contributes to the shedding of meprin β from the cell surface, possibly forming a feedback loop to control cell-surface activity levels of these two metalloproteases.

### Inhibitors

Eight protease inhibitors were identified by TAILS as meprin substrates through identification of meprin cleavage sites (Table 2). These included cystatin C and fetuin-A, which we have previously shown to be endogenous inhibitors of meprin α, with fetuin-A also an inhibitor of meprin β [51] (Online resource 2).

**Table 2:**
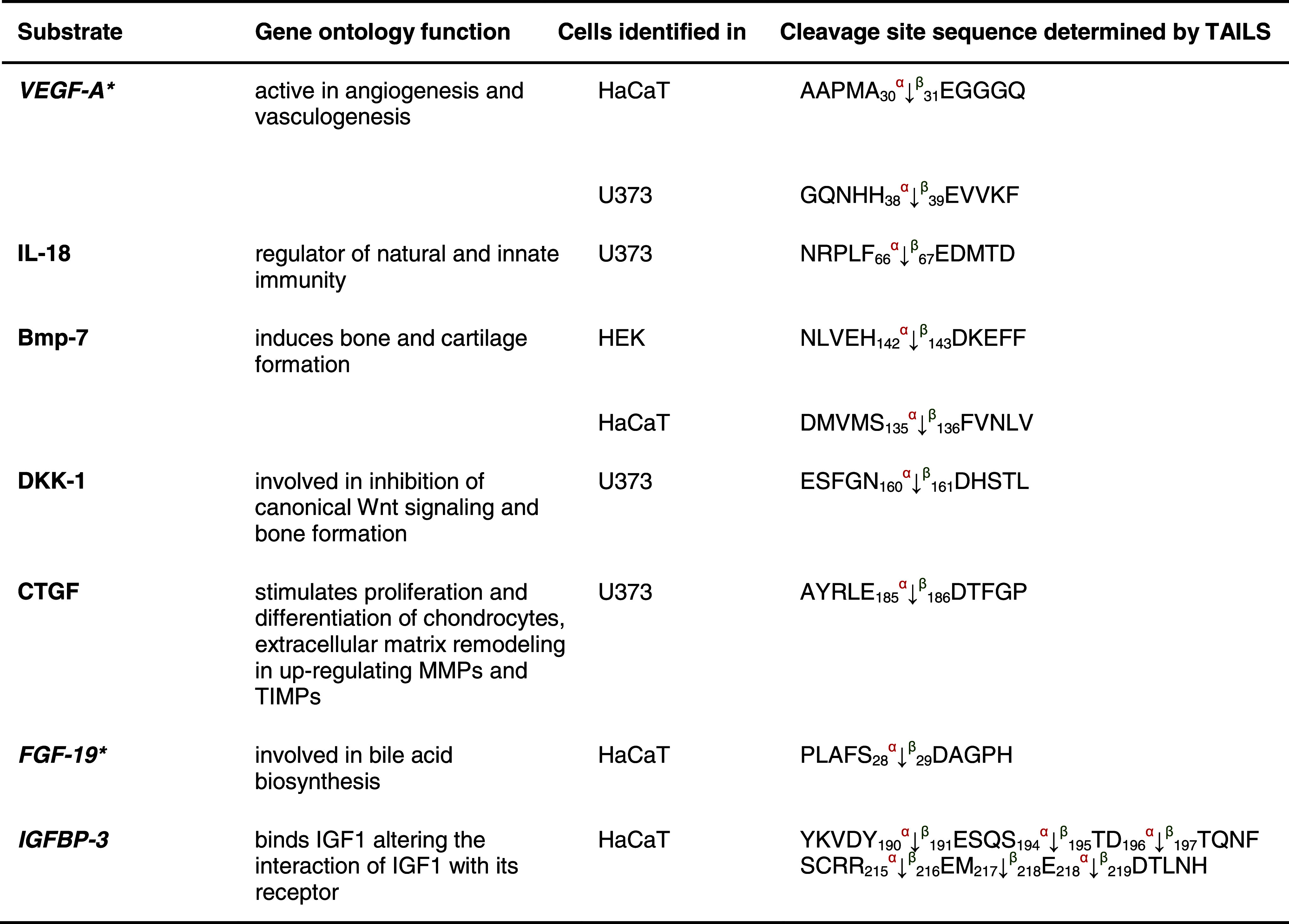
Inhibitor substrates for meprin α and β identified by TAILS

Cystatin C and fetuin-A identified in the conditioned media are known substrates; the others presented are new. Cleavage of cystatin C by meprin α at position Asp^41^↓Ala^42^ was identified in HaCaT and U373 cells. Serpin A3 was hydrolyzed in HaCaT and U373 cells at identical sites. For clusterin, cleavage at position His^263^↓Ser^264^ by meprin β was confirmed in U373 and HaCaT cells. Sequences are given in the *one letter code*. *Arrows* indicate the cleavage sites analyzed by MS/MS for meprin α (*α*) and meprin β (*β*), *asterisks* indicate substrates validated by Edman sequencing. Proteins in *italic* have been further validated in vitro (Online resource 2). *Numbers* indicate the position of amino acids in full-length protein. Confirmed cleavage sites in more than one secretome are indicated in *bold*.

*LEKTI* lymphoepithelial Kazal-type-related inhibitor, *Serpin* serine protease inhibitor.

**Fig. 5:**
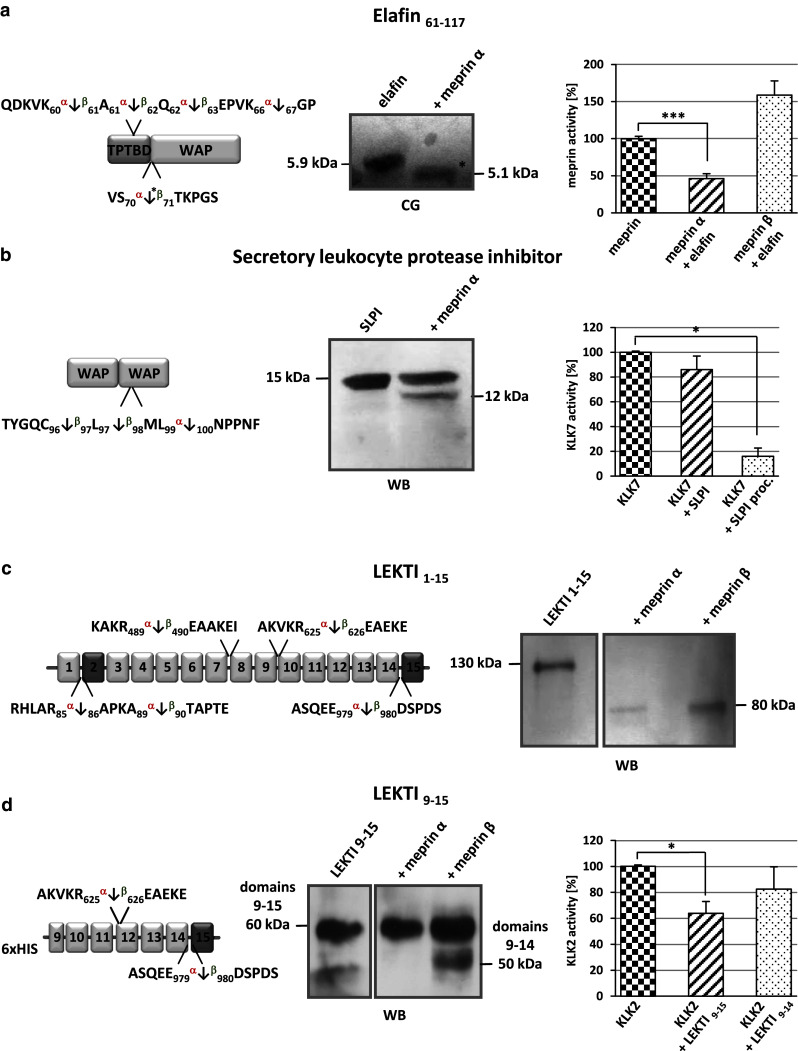
Inhibitors identified by TAILS as substrates for meprin α and β. **a** Elafin processed by meprin α was subjected to SDS-PAGE, transferred to a PVDF membrane and visualized by coomassie blue (*CB*) staining. The cleavage site identified by TAILS is indicated by the *asterisk*. The presence of elafin (5 × 10^−5 ^M) leads to a significant reduction in meprin α activity measured by Mca-YVADAPK(Dnp)-NH_2_ cleavage. Significance was determined by the *t* test (***P < 0.001). **b** Cleavage of SLPI by meprin α was demonstrated by western blot (*WB*) analysis revealing a 12-kDa band. Incubation of KLK7 with meprin α-processed SLPI (*SLPI proc.*) resulted in increased inhibition compared to incubation with untreated SLPI. **c**, **d** Processed fragments of full-length LEKTI 1–15 and LEKTI 9–15 were detected by western blotting (*WB*) using a polyclonal LEKTI antibody. The full-length protein was cleaved by meprin α and β, both releasing an 80-kDa fragment. Specific processing of LEKTI 9–15 by meprin β resulted in a shift from 60 to 50 kDa, matching the TAILS data. Incubation of KLK2 with LEKTI 9–15 resulted in lower proteolytic activity than incubation with meprin β-cleaved LEKTI 9–14

Five cleavage events within the inhibitor elafin were determined by TAILS analysis, all five of which were within the trappin protein transglutaminase binding domain (TPTBD) at position Gln^56^/Asp^57^ by both meprins, which is not present in the recombinant protein we used to validate these proteomic findings, and at Lys^60^/Ala^61^ by meprin α, and at Ala^61^/Gln^62^, Gln^62^/Glu^63^ and Ser^70^/Thr^71^ by meprin α and β. Cleavage at Lys^66^/^67^Gly was due to meprin α cleavage only (Fig. 5a). Ser^70^/Thr^71^ is a cleavage site N-terminal of the whey acidic protein-type (WAP-type) domain, suggesting that this might modulate its antimicrobial activities [54]. Edman degradation was used to validate this cleavage in vitro and the N-terminal sequence exactly matched that obtained in the TAILS data. Thus, even though other proteases and inhibitor activity levels were modified by meprin activity, it is highly likely that in the cell culture experiments the TAILS data reflected direct action of meprin. Analysis of the inhibitory potential of elafin revealed inhibition of meprin α but not of meprin β (Fig. 5a).

TAILS also revealed that SLPI is processed by meprin α and β within its second WAP domain. We validated meprin α cleavage of SLPI in an in vitro assay, by SDS-PAGE and by FRET analysis (Fig. 5b). Notably, cleavage by meprins occurred at the reactive bond of this inhibitor with Leu^97^and Met^98^ being critical for inhibition of chymotrypsin and elastase. Interestingly, SLPI is also cleaved by MMP14 determined previously by ICAT proteomics in the cellular context [55], that showed inactivation of SLPI. Surprisingly, cleavage of SLPI by meprin α significantly increased the inhibitory capacity of this molecule towards the serine protease KLK7 (Fig. 5b).

Another serine protease inhibitor, LEKTI, was shown by TAILS to be cleaved by both meprin α and β (Fig. 5c, d). LEKTI consists of 15 potential inhibitory domains, of which two resemble Kazal-type inhibitors and the other 13 are structurally related but lack one of the three typical disulfide bridges [56, 57]. Western blot analysis showed processing of full-length LEKTI mediated by both meprin α and meprin β that resulted in a shift from 130 to 80 kDa on SDS-PAGE gels (Fig. 5c). According to the TAILS data, cleavage of the last domain of the recombinant LEKTI construct (9–15) by meprin β specifically led to a loss of about 10 kDa in mass (Fig. 5d). The activity of KLK2 after incubation with LEKTI 9–15 mediated by meprin β was about 20 % higher than after LEKTI 9–15 pretreatment due to the presence of the additional domain. We identified two further cleavage sites in LEKTI with ion cut-off ratios of <3.0 between Arg^489^/Glu^490^ and Arg^625^/Glu^626^ in domains 9 and 10, respectively. Processing by either meprin α or β between domains 7 and 8 with additional cleavage of domain 15 resulted in a cleaved protein with a predicted size of 80 kDa.

### Growth factors

**Fig. 6:**
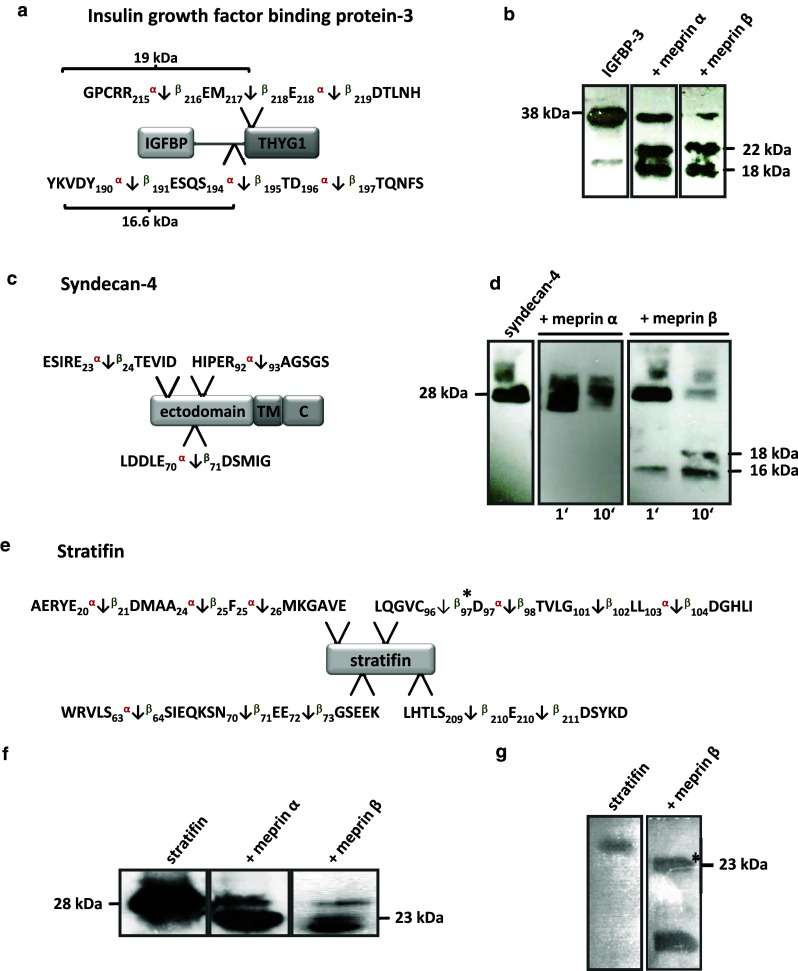
**a** IGFBP-3 identified by TAILS as a substrate for meprin α and β with cleavage sites shown. **b** A shift from 38 kDa to 22 kDa and 18 kDa in processed IGFBP-3 by meprin α and β was demonstrated by western blotting. Molecular masses are higher than their theoretical sizes as indicated by curly braces due to three glycosylation sites at Asn^116^, Asn^136^, and Asn^199^. The sizes of the meprin β-mediated fragments match those expected from the TAILS proteomics data while for meprin α only one cleavage event was detected. **c** Syndecan-4 was identified by TAILS as a substrate for meprin α and β. d Time-dependent processing of syndecan-4 by meprin β revealed a specific cleavage pattern using an antibody for western blotting directed to the His-tag. Cleavage by meprin α resulted in degradation of full-length syndecan-4. e Biochemical validation of stratifin as a meprin substrate. **f** After incubation of recombinant stratifin with meprin α and β, cleavage fragments were transferred to a PVDF membrane and visualized after western blotting with a specific stratifin antibody. The sizes of the cleavage products derived from meprin α and β both matched those expected from the TAILS sequence data. **g** The processed 23-kDa meprin β fragment was subjected to Edman sequencing and confirmed the TAILS-determined cleavage site exactly

We identified fibroblast growth factor 19 and the growth factor associated insulin growth factor binding protein 3 (IGFBP-3) as substrates for both meprin α and β (Table 3). Western blotting confirmed that IGFBP-3 (38 kDa) was cleaved by both meprin α and β (Fig. 6a, b) to two fragments of 22 and 18 kDa, in agreement with size predictions from the TAILS analysis (Fig. 6a, b).

**Table 3:**
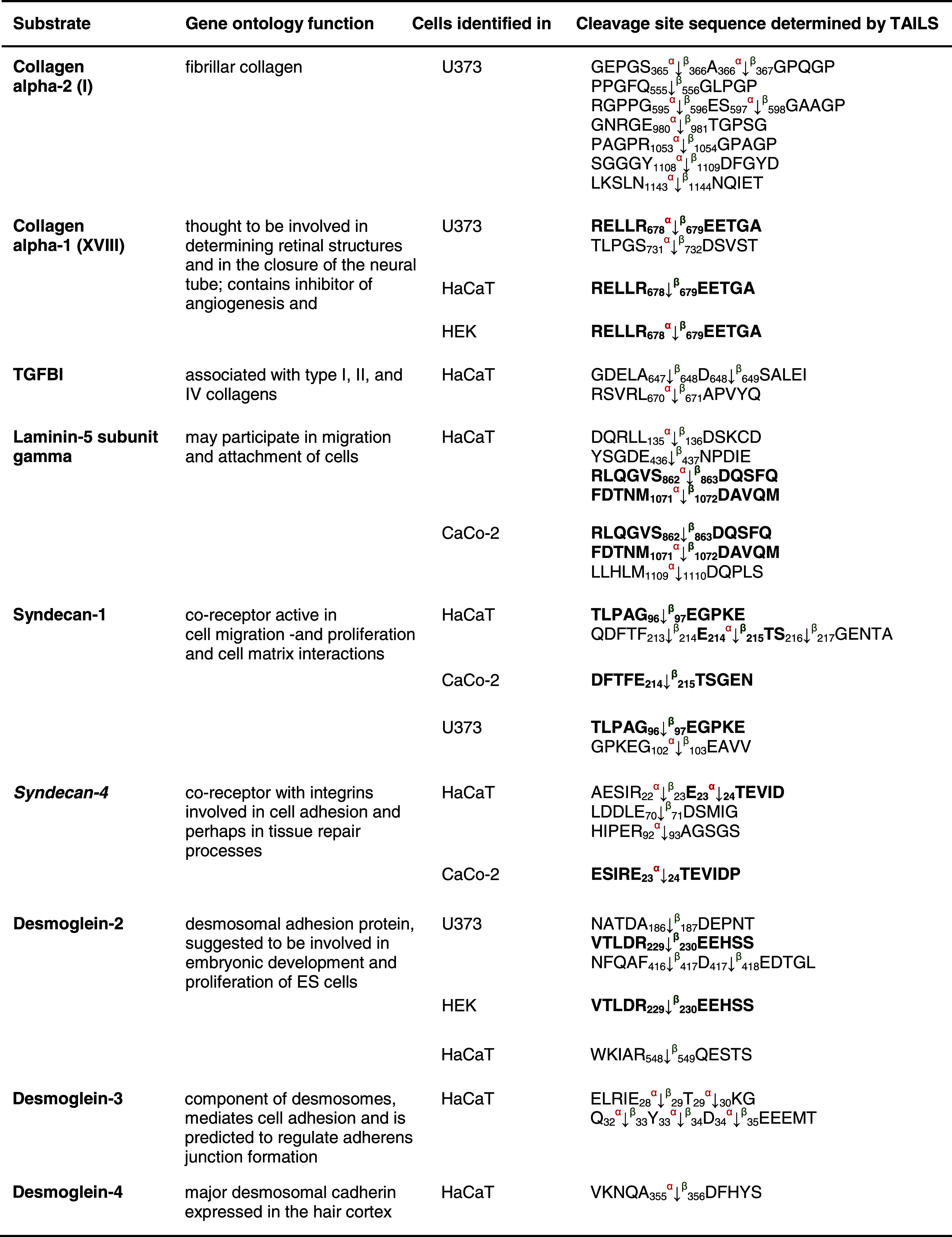

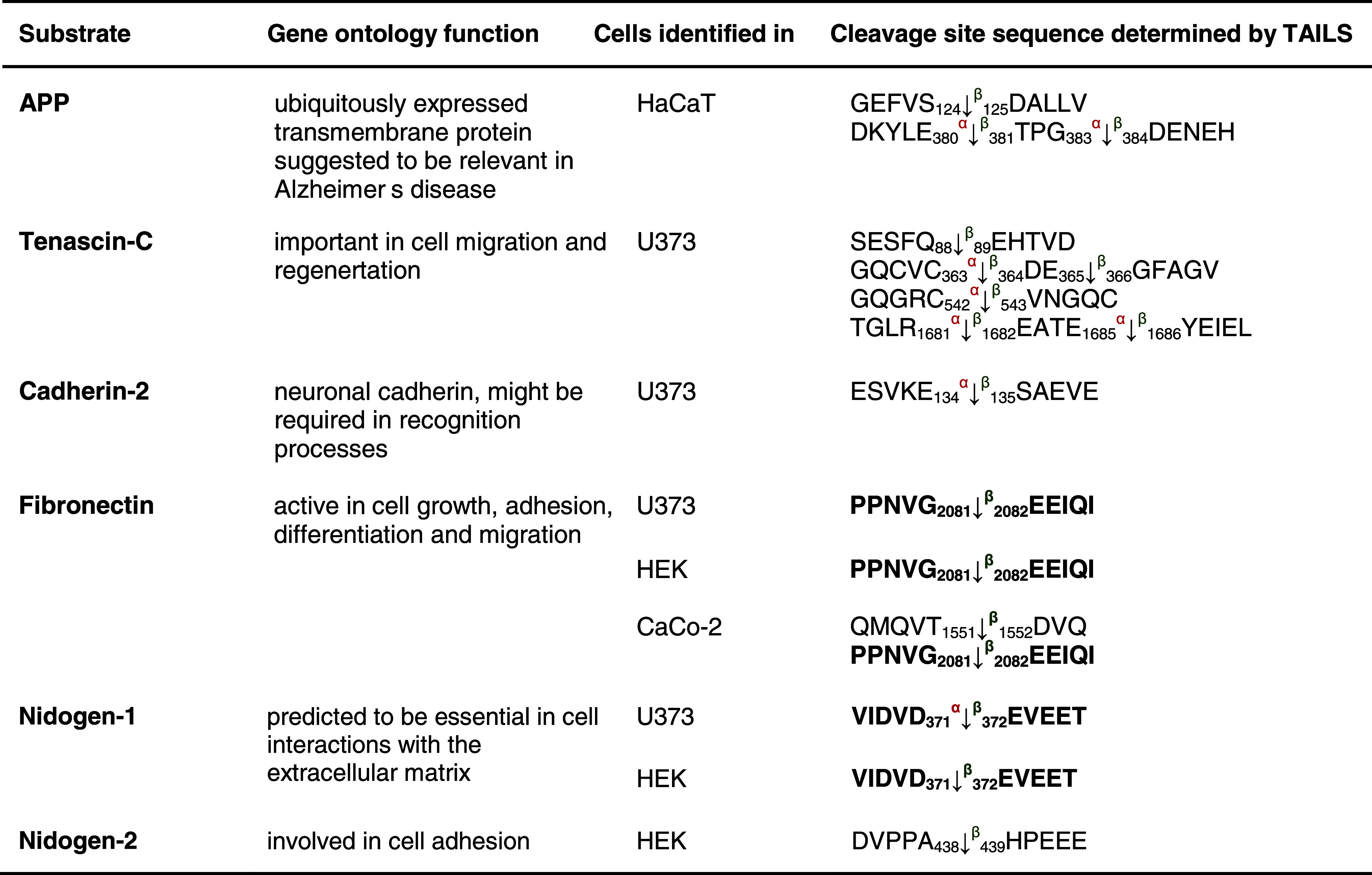
Growth factor candidate substrates for meprin α and β identified by TAILS

Vascular endothelial growth factor A (*VEGF-A*) and interleukin 18 (*IL-18*) are known substrates detected by TAILS as substrates within the class of growth factors; the others are novel substrate candidates. Proteins in *italic* have been further validated in vitro (Fig. 6). Sequences are given in the *one letter codes*. *Arrows* indicate the cleavage sites analyzed by MS/MS for meprin α (*α*) and meprin β (*β*), *Numbers* display position of amino acid in full-length protein. Confirmed cleavage sites in the protein and identified in more than one secretome (biological replicate) is indicated in *bold*.

*BMP-7* bone morphogenic protein 7, *DKK-1* Dickkopf-related protein 1, *FGF-19* fibroblast growth factor, *IGFBP-3* insulin growth factor binding protein 3, *IGF* insulin-like growth factor 1, *MMP* matrix metalloproteases, *TIMP* tissue inhibitor of metalloproteases.

### Extracellular matrix proteins

Both meprin α and meprin β can be released from the cell membrane to the extracellular space [8] where they can cleave collagen IV [58] and procollagen III [15]. We identified two additional substrates from the collagen family for both meprins (Table 4). Collagen α-2 (I) is cleaved by meprin α and/or β N-terminally at Ser^356^, Gly^367^, Gly^556^, Glu^596^, Thr^981^, Gly^1054^, Asp^1109^ and Asn^1144^. Neo-N terminal peptides of collagen α-1 (XVIII) commence at Glu^444^, Glu^679^ and Asp^732^ representing the P1′ residues in the cleavage site. TTGF-β-induced protein ig-h3 (TGFBI), an adhesion molecule that binds to type I fibrillar collagen [59] and is associated with bone formation, was also identified by TAILS as a meprin substrate (Table 5).

**Table 4:**
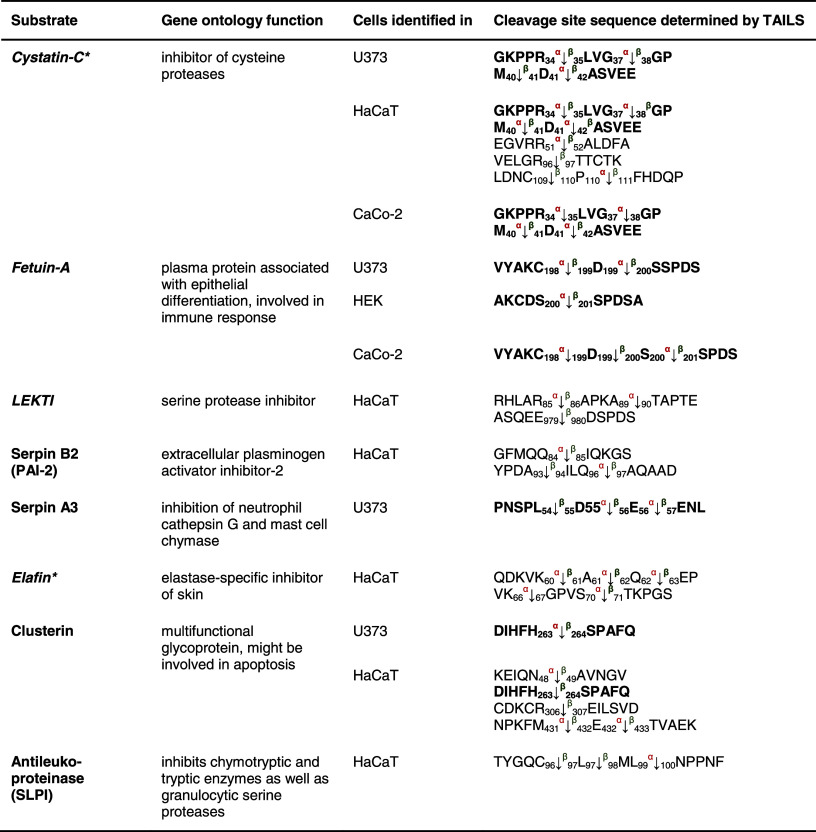
Candidate substrates from the extracellular matrix for meprin α and β identified by TAILS

Protein samples were collected as indicated in Table 1. Except for laminin 5 and the APP, which are known substrates and were also identified by TAILS, the other substrates presented are new candidates for meprin metalloproteases. Proteins in *italic* have been further validated in vivo (Online resource 3). Sequences are given in the *one letter codes*. *Arrows* indicate the cleavage sites analyzed by MS/MS for meprin α (*α*) and meprin β (*β*). *Numbers* display position of amino acid in full-length protein. Confirmed cleavage sites in more than one secretome (biological replicate) are indicated in *bold*.

*TGFBI* transforming growth factor-beta-induced protein ig-h3, *AD* Alzheimer′s disease.

**Table 5:**
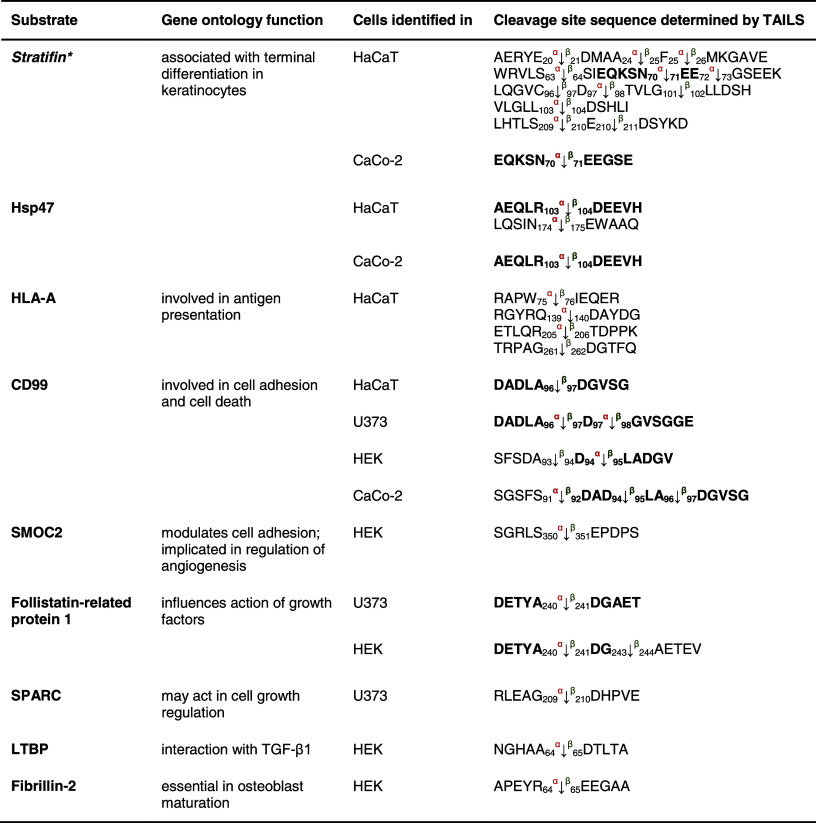
Other substrates for meprin α and β identified by TAILS

Substrates shown here have not been further biochemically validated. Sequences are given in the *one letter code*. *Arrows* indicate the cleavage sites analyzed by MS/MS for meprin α (*α*) and meprin β (*β*). *Numbers* display position of amino acids in full-length proteins. Confirmed cleavage sites in the protein and identified in more than one secretome (biological replicate) are indicated in *bold*.

*HSP* heat-shock protein, *HLA-A* HLA class I histocompatibility antigen, *CD99* cluster of differentiation, *SMOC2* SPARC-related modular calcium-binding protein 2, *SPARC* secreted protein acidic and rich in cysteine, *LTBP* latent-transforming growth factor β-binding protein.

The collagen-binding HSP47 (Table 5), a member of the serpin family and serving as a chaperone [60], was detected as a novel substrate for meprin α and β cleaving between Arg^103^/Asp^104^ and Asn^174^/Glu^175^. Several known substrates of meprin α and β, namely the collagen-binding proteins nidogen-1, nidogen-2, laminin-5γ and fibronectin (Table 4) [58, 61, 62], were identified in the TAILS analysis again showing the power of TAILS to identify substrates.

Several proteoglycans were identified as substrates by TAILS, e.g. syndecan-1 and -4 (Table 4) (Fig. 6). Processing of the 28-kDa syndecan-4 was confirmed when two fragments derived from meprin β with molecular masses of 18 and 16 kDa were detected using a monoclonal antibody specific for the C-terminal His-tag of recombinant syndecan-4 (Fig. 6c, d). The sizes of these cleavage fragments are consistent with cleavage at Glu^70^/Asp^71^ as identified by the neo-N-terminal peptide identified by TAILS (Fig. 6c, d). In contrast, meprin α led to degradation yielding several proteolytic fragments (Fig. 6d). Although meprin β cleaved both proteins in vitro (Fig. 6c, d), only syndecan-4 was identified as a substrate for meprin β by TAILS. This may be because the protein was fully degraded in culture or because the cleavage site lies too close to or too far from an arginine so that the neo-N-terminal peptide is either too short or too long, respectively, for MS identification. Alternately, in a cellular context or in the presence of other molecules, not every potential cleavage site is accessible to the protease.

Finally, the neuronally relevant cadherin-2 and tenascin C were also identified as substrates by TAILS. Tenascin C has recently been found to be cleaved by meprin β and possibly to be involved in Crohn’s disease [63].

### Other substrates

Stratifin, a keratinocyte-specific 14-3-3 protein, was demonstrated to be a substrate for meprin β in TAILS, and this was confirmed in vitro (Fig. 6e–g). Processing by meprin β was much more efficient than that by meprin α, but both yielded a 23-kDa fragment (Fig. 6f). By N-terminal sequencing, the identified meprin β cleavage site between Cys^96^ and Asp^97^ corresponded exactly with that identified by TAILS (Fig. 6g). Table 5 lists additional substrates for meprin α and β that were identified by neo-N-terminal peptides with high iTRAQ ratios.

In total, 151 substrates were identified with high confidence by TAILS, and notably those with cleavage fragments sequenced by Edman degradation in in vitro validation experiments exactly matched the TAILS data. The many examples of this presented here confirms the fidelity of TAILS as a reliable terminomics approach for identifying substrates of proteases, particularly those having a loose cleavage site consensus sequence that makes manual parsing of data unreliable for substrate finding.

## Discussion

Limited proteolytic processing is a crucial molecular mechanism affecting regulation of almost every protein function [22, 23, 64]. Proteases and their endogenous inhibitors build complex dynamic proteolytic systems that are highly interconnected with the signaling networks of chemokines, cytokines and growth factors. To understand this regulatory impact of proteases, whose action is executed by proteolytic activation, it is necessary to systematically elucidate their substrates and the specific cleavage sites within the proteins [4]. Using TAILS, we identified with high confidence 151 extracellular meprin α and β substrates demonstrating an unexpectedly large and diverse substrate degradome for these two proteases. With 33 substrates only reported for human meprins in MEROPS, the protease database [65], and our confirmation of 10 of these known substrates, the identification of a large number of novel substrates by TAILS (141), many of which we validated biochemically, provides a considerable new insight into the in vivo functions of both meprin α and meprin β that until recently have been somewhat enigmatic.

Loss of one protease can raise or decrease the activity of others either directly through altering the activation cascade, shown here for ADAM10, or indirectly by cleaving and removing the activity of a protease inhibitor from the system and hence increasing activity of its cognate protease, as we showed for the inhibitors LEKTI, SLPI, elafin and cystatin C, for example. Previous data have shown that meprins are involved in the progression of several pathological conditions, but the substrates in these diseases are largely unknown [8]. Our findings reveal proteolytic molecular interactions that link to possibly explaining some of these events. We demonstrated that meprin β is an activator of ADAM10, a metalloprotease known to be important for development and tissue maintenance, mediated by Notch or EGFR ligand shedding [20]. The lack of meprin in knockout mice is correlated with aggravated chronic inflammation, but specific substrates have been elusive [66]. The reduced ADAM10 activity found in meprin β knockout mice would clearly have impact on inflammatory processes such as inflammatory bowel disease, since it has been shown that ADAM10 cleaves inflammatory cytokines [67]. However, such an association between a protease and its activator also renders it difficult to ascribe the murine phenotype to either protease without further experimentation [68].

ADAM10 is synthesized as a zymogen requiring proteolytic activation. Although proprotein convertase PC7 and furin are known to process the propeptide at the maturation site RKKR, a second cleavage event is necessary to release mature ADAM10 from the prodomain [35]. However, the responsible proteolytic mechanism has been elusive. Our data show that the meprins can activate ADAM10 to completion not only in in vitro experiments, but also in the cellular context. The propeptide is required for proper protein folding and secretion of the catalytic domain [69], but is also bound in a noncovalently enzyme–propeptide complex, thereby inhibiting ADAM10 activity [35]. Isolated murine ADAM10 propeptide, for example, selectively inhibits the catalytic domain of human ADAM10 in the nanomolar range, both in vitro and in cellulo, and in micromolar concentrations blocks the activity of other ADAM family members [35].

The activation of ADAM10 by meprin β is of special interest in relation to the development of Alzheimer′s disease. Our findings suggest that increased α-secretase activity mediated by meprin β potentially prevents progression of Alzheimer’s disease. We are presently initiating further investigations with the aim of proving this possibility, but in support of this hypothesis, we found altered proteolytic processing of the APP in meprin β-expressing mice compared to the animals lacking this protease (Online resource 3).

ADAMs are also important for the shedding of proteolytic enzymes from the plasma membrane [20, 70, 71]. ADAM10 itself is released from the cell surface through cleavage by ADAM9 and ADAM15, indicating that ADAM10 has dual functions in the cell [72].

The membrane-associated meprin β is shed from the cell surface by ADAM17 (TACE) cleaving N-terminal to the epidermal growth factor-like domain upon PMA stimulation [3]. In our work, we showed ADAM10-mediated meprin β shedding in cellulo, although ADAM17 was found to be the major releasing enzyme. In contrast to the membrane-tethered form, release of meprin β modifies access to different substrates depending on cellular or extracellular location. This is pertinent to meprin substrates located at the cell surface, as known for APP [16]. The release of cell surface proteases such as ADAMs and meprin β by ectodomain shedding opens a fourth dimension (time) in proteolytic signaling that has to be taken in account in further studies.

Consistent with the new network connections we found between meprins and ADAMs, we found several cleavage events that do not fit with the strong preference for negatively charged amino acids in P1′ [14]. The activity of ADAM10 and KLK7 increases after meprin cleavage, and so these proteases may contribute to the cleavage we identified in the secretomes. Cleavage specificities for KLK7 by positional scanning of the positions P1–P4 revealed that the chymotryptic protease favors tyrosine in P1 and hydrophobic amino acids in P2 [73]. Hence, the availability of the specificity profiles of other proteases allows determination of whether an identified TAILS substrate is a real meprin substrate or appears as a downstream effect. For example, ADAM19 (Tyr^92^/Thr^93^) and the serum amyloid A protein (Tyr^47^/Ile^48^) show a typical chymotryptic cleavage site with tyrosine in P1 indicating that processing most likely takes place as a secondary event and is not based on direct meprin cleavage.

Cleavage of desmoglein-2 at Arg^548^/Gln^549^ also does not match the canonical pattern for meprins, and desmoglein-2 is a known substrate of KLK7 and other KLKs under (patho)physiological conditions [74]. Hence, both the identified cleavage sites are probably due to effects downstream from meprin activity. Serine protease 23 (Pro^23^/Tyr^24^) and the beta-2-microglobulin (Cys^45^/Tyr^45^) also exhibited cleavage sites not typical of meprins. ADAM10 has been shown to promote high selectivity for Tyr in P1′ [75] making the two substrates more likely due to ADAM10 activity as a downstream effect.

Regulation of protease activity is also mediated by endogenous inhibitors such as the four TIMPs for MMPs and TIMP3 for ADAMs, which is crucial for the orchestration of proteolytic signaling [23]. Recently, we demonstrated that fetuin-A is an inhibitor of several astacins including meprin α and β with an inhibition constant (*K*_i_) of 4.2 × 10^−5^ M and 1.1 × 10^−6^ M, respectively [51]. Here, we identified fetuin-A and cystatin C as substrates for both meprins by TAILS (Online resource 2). Concerning fetuin-A, the cleavage pattern after incubation with meprin α was different than that with meprin β. With regard to the inhibitory potential of fetuin-A and cystatin C, kinetic studies indicated that cystatin C is a relevant meprin α inhibitor, whereas fetuin-A exhibits stronger inhibition of meprin β. We also evaluated the potential of elafin as an inhibitor of meprin α which has an inhibition mechanism similar to that of cystatin C. Meprin α was significantly inhibited by elafin while meprin β exhibited increased activity. The possibility that the inhibitory capacity was simply due to concurrent substrate effects was excluded in our previous study in which we evaluated meprin activity in the presence of a large excess of the known substrate gelatin, and found no significant difference [51]. It seems likely that meprin inhibition by these molecules is initiated by proteolytic cleavage. However, only crystallization of an enzyme/inhibitor complex could reveal appropriate information.

Cleavage of cystatin C by MMPs leads to inactivation and a subsequent increase in cathepsin L activity [26]. Cystatin C was not simply degraded, but also inhibited meprin α, whereas cleavage by meprin β rather resembled the more selective MMP processing. Some protein inhibitors appear to need an initial processing event to gain inhibitory maturity, while others slip into inactivity [76, 77]. In cystatin C, two cleavage sites for meprin α at Arg^34^/Leu^35^ and Leu^36^/Val^36^ were detected by TAILS. These have been previously found in urine from nephrology patients [78]. The cleavage occurs one residue C-terminal of the known reactive site for cystatin C, and therefore may be decisive for meprin inhibition. Hence, meprin expression and activity probably correlate with the emergence of pathological disorders such as nephritis in which meprin is upregulated [79, 80].

Interestingly, both meprin α and meprin β were able to cleave the plasminogen activator inhibitor-2 (PAI-2 or serpin B2) (Table 4). In the skin, PAI-2 has been shown to be a substrate of transglutaminase-1 and to be crosslinked into the cornified envelope [81]. Given that meprin β is expressed in the stratum granulosum of the epidermis [40], it is likely that under physiological conditions only meprin β cleaves PAI-2. However, in Netherton syndrome, in which meprin α is also detected in the stratum granulosum, proteolytic interaction with PAI-2 might contribute to the progression of this hyperproliferative condition.

Two members of the trappin family, SLPI and elafin, and LEKTI are present in human epidermis and may inhibit the serine protease KLK7 to control downstream proteolytic cascades [82]. Interestingly, SLPI is cleaved at the Leu-Met bond, which is critical for inhibitory activity, but we one found first for MMP14 at this same site in SLPI, revealing a common control point in serine protease activity by metalloprotease inactivation of a serine protease inhibitor [55]. Dysfunction of the KLK7 inhibitors mediated by meprins might lead to increased degradation of desmoglein-1 and subsequently enhanced desquamation of the skin. Moreover, zymogenic proKLK7 was also identified as a meprin substrate, where cleavage occurred two amino acids N-terminal from the activation site (Online resource 4). Recently, we have shown that this primary meprin β-mediated processing results in accelerated activation of proKLK7 [83].

To close a feedback circle, meprin β-mediated ADAM10 activation might also be important for skin homeostasis, since it has been demonstrated that ADAM10 activity is essential for epidermal integrity [30]. Thus, dysregulated meprin activity, due to expression, activation, shedding or inhibition, would lead to altered activities of ADAM10, KLK7 and LEKTI, which are all important for skin homeostasis. Thus, our proteomic analyses and biochemical studies have provided several new insights into the biological role of meprins in skin that we are presently investigating further.

Both meprin α and meprin β process growth factors, cytokines and hormones [12, 84–86]. Meprin β activates the cytokine IL-18 in mice, cleaving between Asn^51^ and Asp^52^ [10]. TAILS also showed cleavage of human IL-18 at position Phe^66^/Glu^67^ (Table 3), confirming the findings of the previous studies. Among the identified growth factor substrates, of particular interest is VEGF-A (Online resource 3). Morpholino knockdown of meprin α in zebrafish embryos has revealed that this protease plays an important role in the correct formation of the vascular system [12] (Hedrich et al., in preparation). In developing organisms, VEGF_165_-A plays a major role in angiogenesis and is coexpressed with connective tissue growth factor (CTGF). Immobilization of VEGF_165_-A by complexing with CTGF results in antiangiogenic effects since VEGF-A is prevented from binding to its receptor [87]. However, MMP1 and MMP7, identified here as meprin substrates (Table 1) (Online resource 4), are able to process CTGF complexed with VEGF-A leading to the recovery of VEGF_165_-A angiogenic activity [87, 88]. Meprin α and β process not only MMPs but also CTGF itself (Table 3). Whether cleavage occurs when CTGF is bound in a complex remains to be elucidated, but a study analyzing porcine uterine fluids [89] has demonstrated CTGF cleavage products starting with the same N-terminus (Asp^186^) as we identified for meprin α and β.

**Fig. 7:**
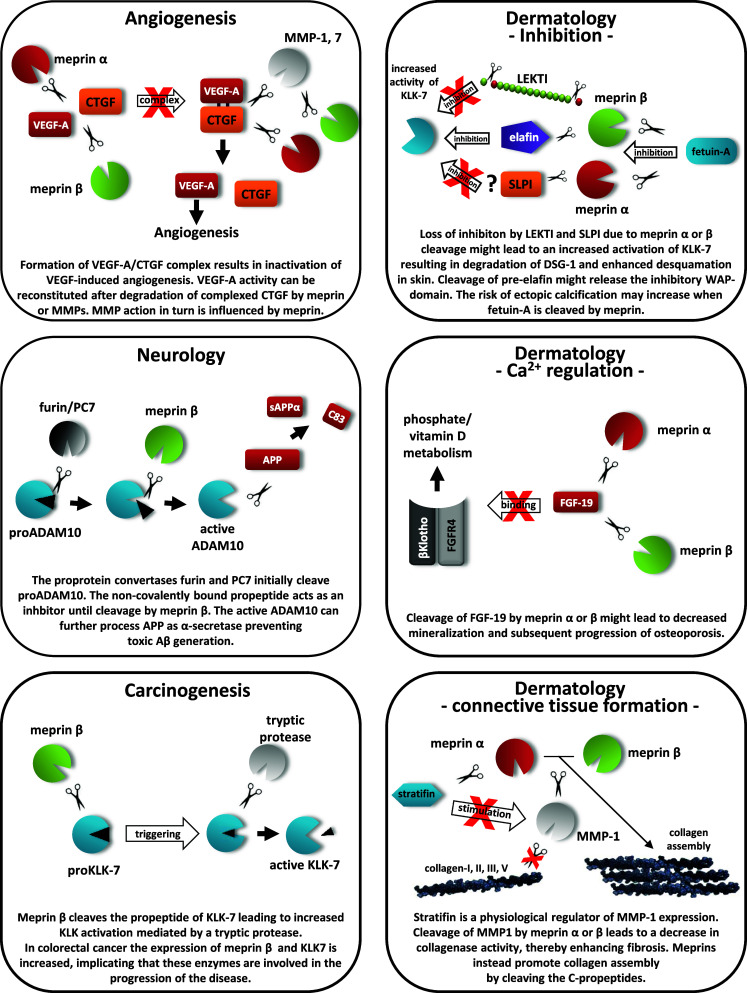
Cleavage of meprin substrates influencing the protease web. The schemes illustrate several substrates of meprin α and β identified by TAILS and show predicted in vivo roles when the substrates are proteolytically processed

In conclusion, we present data that led to the identification of important novel targets for the metalloproteases meprin α and β based on a substrate screen of the entire proteome (Fig. 7). Among the 151 identified extracellular substrates, the findings of activation of several protease zymogens and the inactivation of protease inhibitors reveal that meprins are important nodes in the protease web. Such modifications to the protease web also modify substrate processing in vivo [21–23]. While this renders interpretation of findings from protease knockout mice difficult, and indicates the need for further experimentation to discriminate direct from indirect effects, the very large number of in vitro validations performed here by TAILS analysis (that were exactly backed up by Edman degradation analysis of meprin cleavage sites in several substrates) confirmed that many of the meprin substrates found are of direct biological relevance. This new understanding of meprin substrates indicates important and unexpected new biological roles played by meprins. This, together with knowledge of their substrate degradomes, are important steps in the understanding of related pathological conditions, and subsequently also important for drug development.

### Electronic supplementary material

Below is the link to the electronic supplementary material.

Supplementary material 1 (DOC 1.37 MB)
