# Supplementary material for: The substrate degradome of meprin metalloproteases reveals an unexpected proteolytic link between meprin β and ADAM10
Source: Cell Mol Life Sci. 2012 Sep 1;70(2):309–33. doi: 10.1007/s00018-012-1106-2 (PMC3535375; doi:10.1007/s00018-012-1106-2)
Supplement: Supplementary file 1 — Supplementary material 1 (DOC 1.37 MB) [file 18_2012_1106_MOESM1_ESM.docx]

**Cellular and Molecular Life Sciences**

**The substrate degradome of meprin metalloproteases reveals**

**a proteolytic link between meprin β and ADAM10**

**Running title: TAILS proteomics analysis of meprin α and β**

Tamara Jefferson^1^, Ulrich auf dem Keller^2,3^, Caroline Bellac^3^, Verena V. Metz^4^, Claudia Broder^1^, Jana Hedrich^5^, Anke Ohler^6^, Wladislaw Maier^7^, Viktor Magdolen^8^, Erwin Sterchi^9^, Judith S Bond^10^, Arumugam Jayakumar^11^, Heiko Traupe^12^, Athena Chalaris^1^, Stefan Rose-John^1^, Claus U Pietrzik^7^, Rolf Postina^4^, Christopher M Overall^3^, Christoph Becker-Pauly^1^*

^1^ Institute of Biochemistry, Christian-Albrechts-University, 24118 Kiel, Germany

^2^ Institute of Molecular Health Sciences, Swiss Federal Institute of Technology Zurich, ETH Hoenggerberg, HPM D24, Zurich, Switzerland

^3^ Centre for Blood Research, Departments of Oral Biological and Medical Sciences and Biochemistry and Molecular Biology, University of British Columbia, Vancouver, British Columbia, Canada

^4^ Institute of Pharmacy and Biochemistry, Johannes Gutenberg-University, Mainz, Germany

^5^ Institute of Physiology and Pathophysiology, University Medical Center of the Johannes Gutenberg-University, Mainz, Germany

^6^ Cell and Matrix Biology, Johannes Gutenberg University, Mainz, Germany

^7^ Institute of Pathobiochemistry, University Medical Center of the Johannes Gutenberg-University, Mainz, Germany

^8^ Clinical Research Unit, Department of Obstetrics and Gynecology, Technical University of Munich, Munich, Germany

^9^ Institut of Biochemistry and Molecular Medicine, University of Bern, Bern, Switzerland

^10^ Department of Biochemistry and Molecular Biology, The Pennsylvania State University College of Medicine, Hershey, PA, USA

^11^ Department of Experimental Therapeutics, The University of Texas, M.D. Anderson Cancer Center, 1515 Holcombe Blvd., Houston, TX, USA

^12^ Department of Dermatology, University Hospital Münster, Münster, Germany

*Prof. Dr. Christoph Becker-Pauly Email: cbeckerpauly@biochem.uni-kiel.de

Unit for Degradomics of the Protease Web Phone: 0049-431-880 7118

Christian-Albrechts-University Fax: 0049-431-880 5007

Rudolf-Höber-Str. 1,

24118 Kiel, Germany **Material and methods**

***In vitro scratch-invasion assay of keratinocytes***

The scratch assay was used to measure HaCaT cell migration *in vitro.* Using a sterile pipette tip, a wound was simulated by straight scratching of the cells grown to 90 % confluency in 6-well plates. FGF-19 (0.5 ng/ml) and 0.5 ng/ml FGF-19 incubated with 10^-10^ M meprin β were applied to the injured cell layers. Wound closing was documented at the start of the assay (0 h) and after 27 h using a phase contrast microscope. For each time point the cell free space was calculated in relation to the primary time point.

***Immunoelectron microscopy***

Biopsies of human skin were fixed in 0.1% glutaraldehyde and 3% paraformaldehyde in 0.1 M phosphate buffer (pH 7.4) for 3 h at room temperature. Fixed tissue was dehydrated to 96% ethanol, embedded in LR White hard (Science Services, Munich, Germany), and polymerized at 4°C under ultraviolet (UV) light for 48–60 hours. Ultrathin sections (50 nm) were cut on a Leica Ultracut S and were collected on Formvar-coated nickel grids. Sections were first etched with saturated sodium periodate (Sigma) at room temperature for 2 min. The grids were pre-incubated with 0.1% Tween 20 in phosphate-buffered saline (PBS), then blocked with 50 mM NH4Cl in PBS and in blocking solution (0.5% fish gelatin (Sigma) plus 0.1% ovalbumin (Sigma) in PBS). Sections were incubated with primary antibodies against meprin β diluted in blocking solution at 4°C for 60 h, washed in PBS and twice in a mixture of 0.1% ovalbumin, 0.5% cold-water fish gelatin, 0.01% Tween 20, 0.5 M NaCl in 10 mM phosphate buffer, pH 7.3 (IgGgold buffer). The sections were incubated for 2 h with goat anti-rabbit Fab conjugated to nanogoldTM (1.4-nm gold particles; Nanoprobes, Stony Brook, NY), diluted in IgG-gold buffer. Washed sections were post-fixed in 1% glutaraldehyde for 5 min and air-dried. The nanogoldTM labeling was silver-enhanced for 25 min at room temperature as described (Danscher, 1981). The grids were then washed in distilled water and stained with 2% ethanolic uranyl acetate for 10 min and with lead citrate for 2 min before examination in a FEI Tecnai 12 TEM (Sato, 1968).

**References**

Danscher G (1981) Localization of gold in biological tissue. A photochemical method for light and electronmicroscopy. *Histochemistry* **71:** 81-88

Ishii K (2007) Identification of desmoglein as a cadherin and analysis of desmoglein domain structure. *The Journal of investigative dermatology* **127:** E6-7

Sato T (1968) A modified method for lead staining of thin sections. *J Electron Microsc (Tokyo)* **17:** 158-159

**
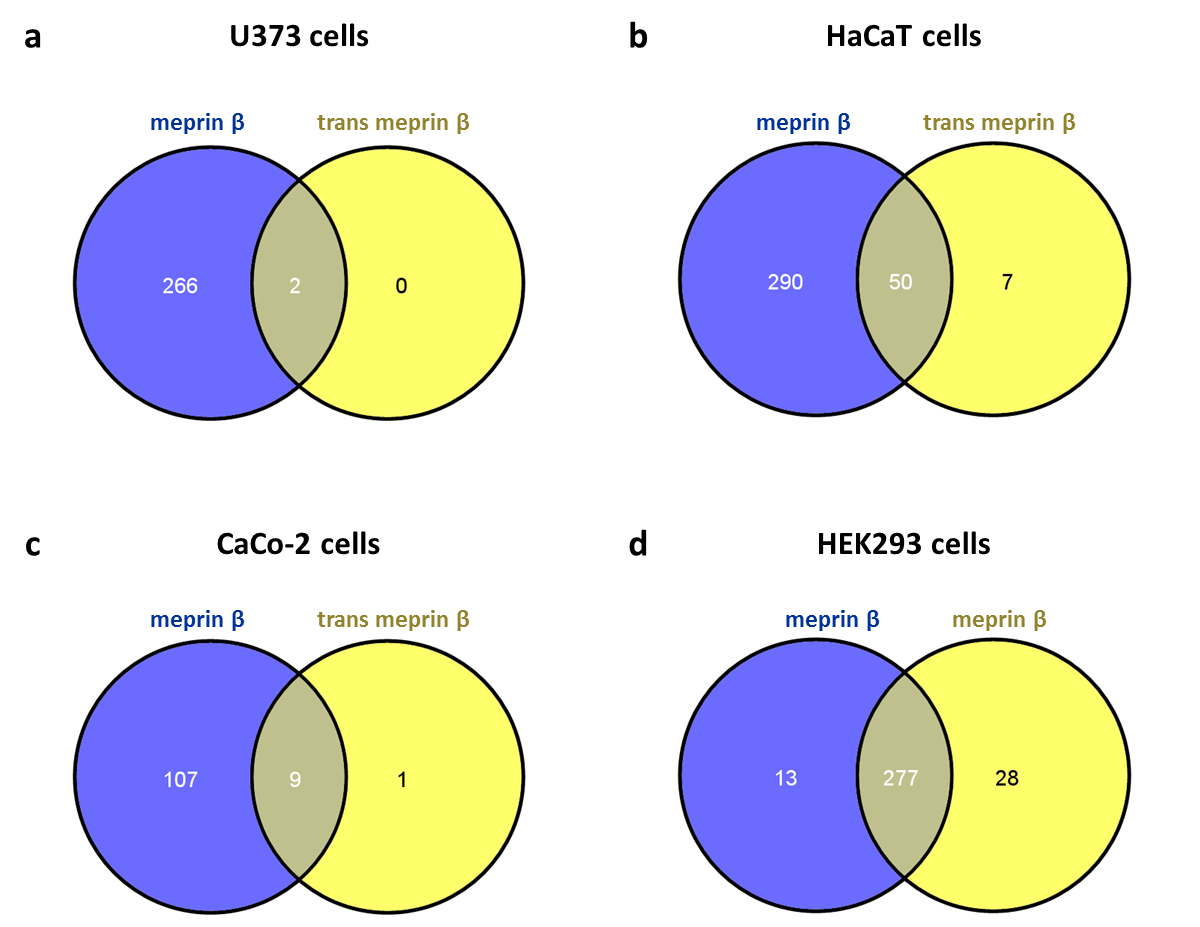
**

**ESM_1** (a-c) Distribution of N-terminal peptide numbers identified with high confidence (ion ratio cut off of 10.0) was compiled in a two way Venn diagram from the neo-N terminal peptides generated by transfection of U373, HaCaT, and Caco2 cells with meprin β (trans meprin β) and application of recombinant meprin β. (d) Reproducibility was confirmed using HEK293 cells incubated with recombinant meprin β in two biological replicates demonstrating high concordance represented by a number of 277 compared to a total of 290 respectively 305 cleavage sites in single samples.

**
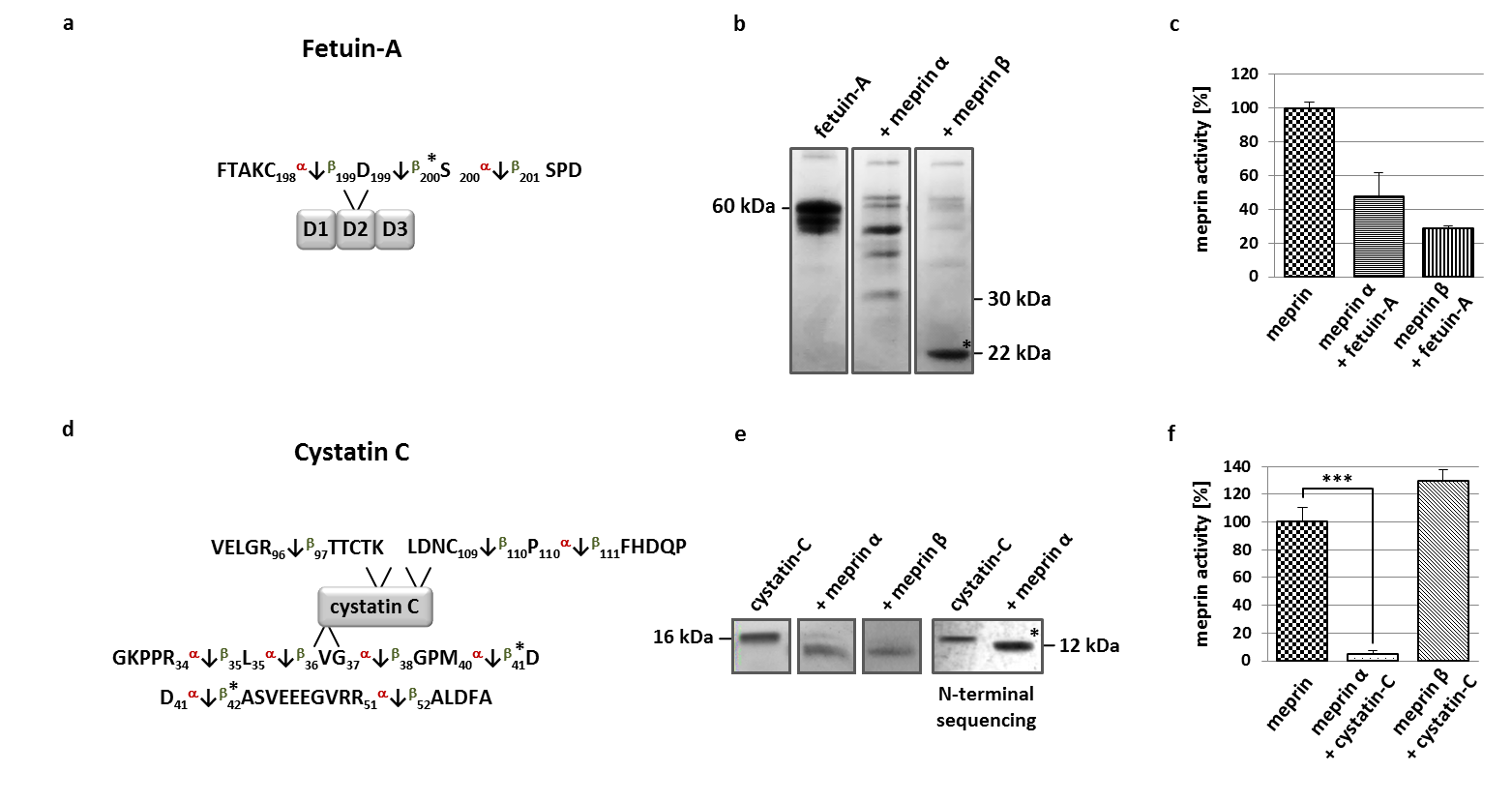
**

**ESM_2** (a, b) Cleavage sites at Cys^198^↓Asp^199^, Asp^199^↓Ser^200^ for meprin β were detected in fetuin-A. Recombinant fetuin-A was incubated with meprin α and β, separated by SDS-PAGE and coomassie stained. Asterisk indicates digested meprin β-derived fragment corresponding exactly to the TAILS determined cleavage site. (C) Meprin α (5 nM) and meprin β (1 nM) were inhibited by fetuin-A (1.89 x 10^-5^ M) as determined with the FRET-substrate Mca-YVADAPK(Dnp)-NH_2_ (R&D Systems)_._

(d, e) Cleavage of cystatin C by meprin α and β was visualized on a coomassie stained SDS-PAGE gel and PVDF membrane. Sequence obtained by Edman degradation is displayed by the asterisk and matched the most C-terminal (larger) fragment. The semi-tryptic peptides generated after trypsin cleavage in the proteomics workflow will be too small or redundant to be identified by mass spectrometry. (f) Meprin α (5 nM) was selectively inhibited by 4.13 x 10^-5^ M recombinant cystatin C while meprin β (1 nM) activity is not inhibited as detected at 405 nm using the FRET substrate Mca-YVADAPK(Dnp)-NH_2_. Significance was determined by the t-test (*** = P < 0.001).

**
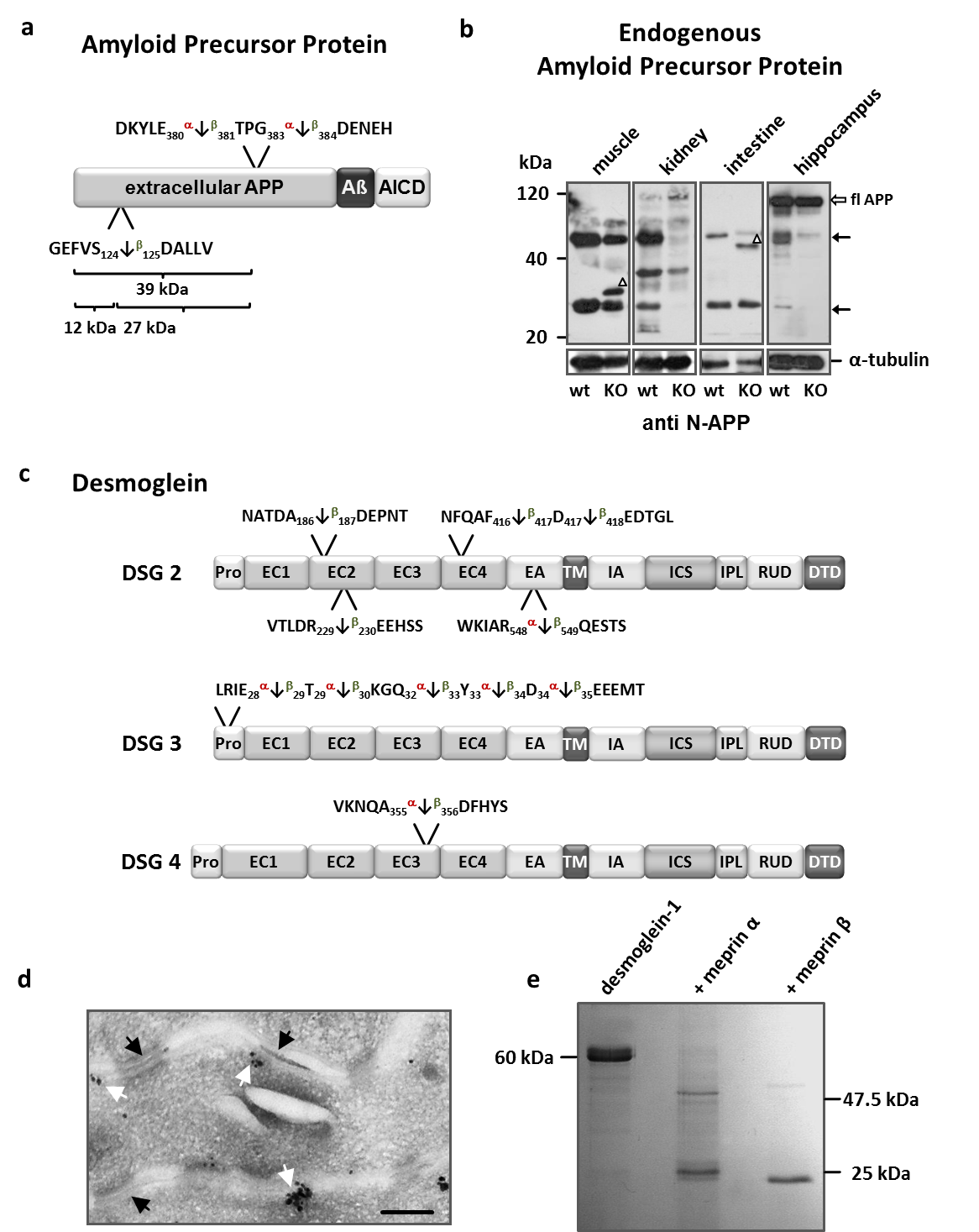
**

**ESM_3** (a, b) Amyloid precursor protein (APP) processing in meprin β^-/-^ mice is altered compared to wild type animals. N-terminal fragments of the amyloid precursor protein detected with the monoclonal anti-N-APP antibody differ between wild type (wt) and meprin β^-/-^ (KO) mouse organs. The triangle marks novel N-APP fragments due to the lack of meprin β activity in muscle and intestine of meprin β^-/-^ mice lysates that were absent in wild type animals. Indicated by the arrows are amyloid precursor protein cleaved forms that are present in every wild type lysate. White arrow indicates the full length amyloid precursor protein (fl APP).

(c) The schematic overview of the three desmogleins-2, 3 and 4 (Ishii, 2007) shows the cleavage sites identified by TAILS for meprin α and β. (d) Localization of meprin β in the epidermis determined by electron microscopy and immunogold labeling with a meprin β-specific antibody revealed co-localization with desmosomes. White arrows indicate desmosomes, black arrows immunogold particles.

Bar = 300 nm. (e) Cleavage of recombinant desmoglein-1 by meprin α resulted in two major fragments of 47.5 and 25 kDa. Processing by meprin β revealed fragments of marginally smaller molecular sizes.

Pro: propeptide; EC1-4: extracellular subdomains; EA: extracellular anchor; TM: transmembrane domain; IA: intracellular anchor; ICS: intracellular cadherin-typical sequence; IPL: intracellular proline-rich linker domain; RUD: repeated unit domains; DTD: desmoglein-specific terminal domain; TM: transmembrane domain; C: C-tail; APP: amyloid precursor protein; Aβ: amyloid β; AICD: APP intracellular domain.

**
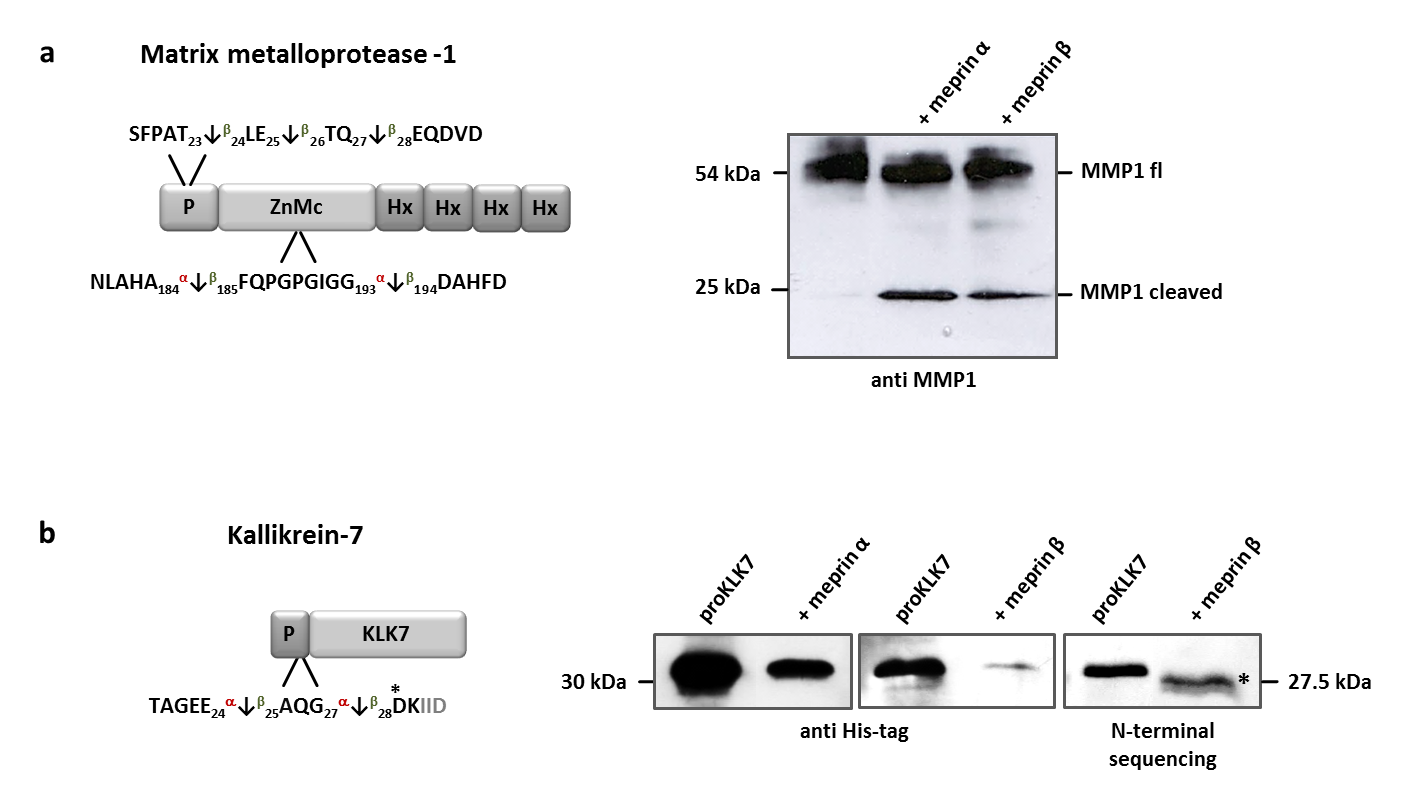
**

**ESM_4** (a) Validation of MMP1 as a substrate for meprin α and β. Following incubation of MMP1 with meprin α and β for 120 min, cleaved MMP1 was separated by SDS-PAGE and the gel coomassie stained. Processing of MMP1 by meprin α and β resulted in the generation of proteolytic fragments matching the TAILS cleavage site. A glycosylation site at Asn^120^ likely leads to a higher molecular weight in the SDS gel resulting in 25 kDa. MMP1: matrix metalloprotease-1; Hx: Hemopexin-like repeats; ZnMc: Zinc-dependent metalloprotease domain; P: prodomain, fl: full-length.

(b) Prokallikrein related peptidase 7 (KLK7) identified as a substrate by TAILS was incubated for 120 min with meprin α and β. Control and cleaved proteins were subjected to SDS-PAGE and visualized by immunodetection with an anti His-tag antibody. Processing of proKLK7 by meprin β was also verified by N-terminal sequencing indicated by the asterisk and the Edman determined sequence matched exactly the neo-N terminal peptide identified by TAILS.

**
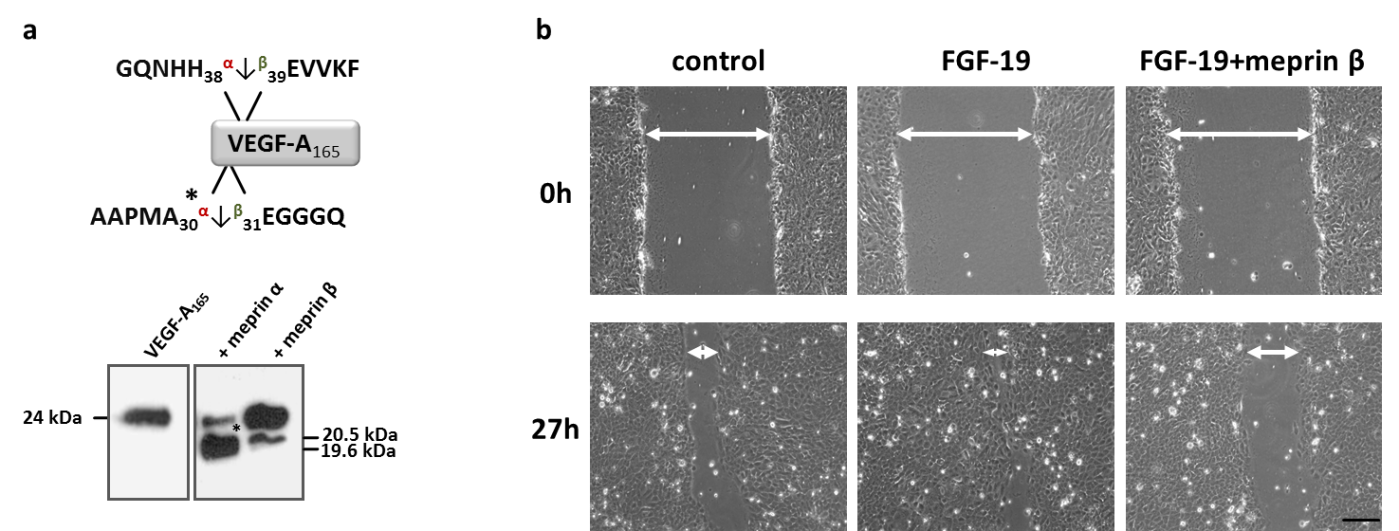
**

**ESM_5** (a) The splicing variant VEGF-A_165_ was cleaved by meprin α and β resulting in a 19.6 kDa product for meprin α and 20.5 kDa for meprin β after 30 min incubation and visualization with an anti-VEGF-A antibody. The cleavage site identified by TAILS corresponds exactly to the sequence retrieved by Edman degradation and is marked by the asterisk. (b) Keratinocytes migration directed by FGF-19. Following scratching of confluent HaCaT cells, recombinant FGF-19 (0.5 ng/ml) and meprin β (10^-10^ M) cleaved FGF-19 were applied and documented at the start (0 h) and after 27 h of assay. Compared to the untreated control and cells incubated with the growth factor alone, cleaved FGF-19 results in a significant delay of migration and wound
